# Supplementary material for: Glucose-Dependent Insulinotropic Polypeptide Receptor-Expressing Cells in the Hypothalamus Regulate Food Intake
Source: Cell Metab. 2019 Nov 5;30(5):987–996.e6. doi: 10.1016/j.cmet.2019.07.013 (PMC6838660; doi:10.1016/j.cmet.2019.07.013)
Supplement: Document S1. Figures S1–S4 [file mmc1.pdf]

Cell Metabolism, Volume *30*

## Supplemental Information

# Glucose-Dependent Insulinotropic Polypeptide

# Receptor-Expressing Cells

# in the Hypothalamus Regulate Food Intake

**Alice E. Adriaenssens, Emma K. Biggs, Tamana Darwish, John Tadross, Tanmay Sukthankar, Milind Girish, Joseph Polex-Wolf, Brain Y. Lam, Ilona Zvetkova, Warren Pan, Davide Chiarugi, Giles S.H. Yeo, Clemence Blouet, Fiona M. Gribble, and Frank Reimann**

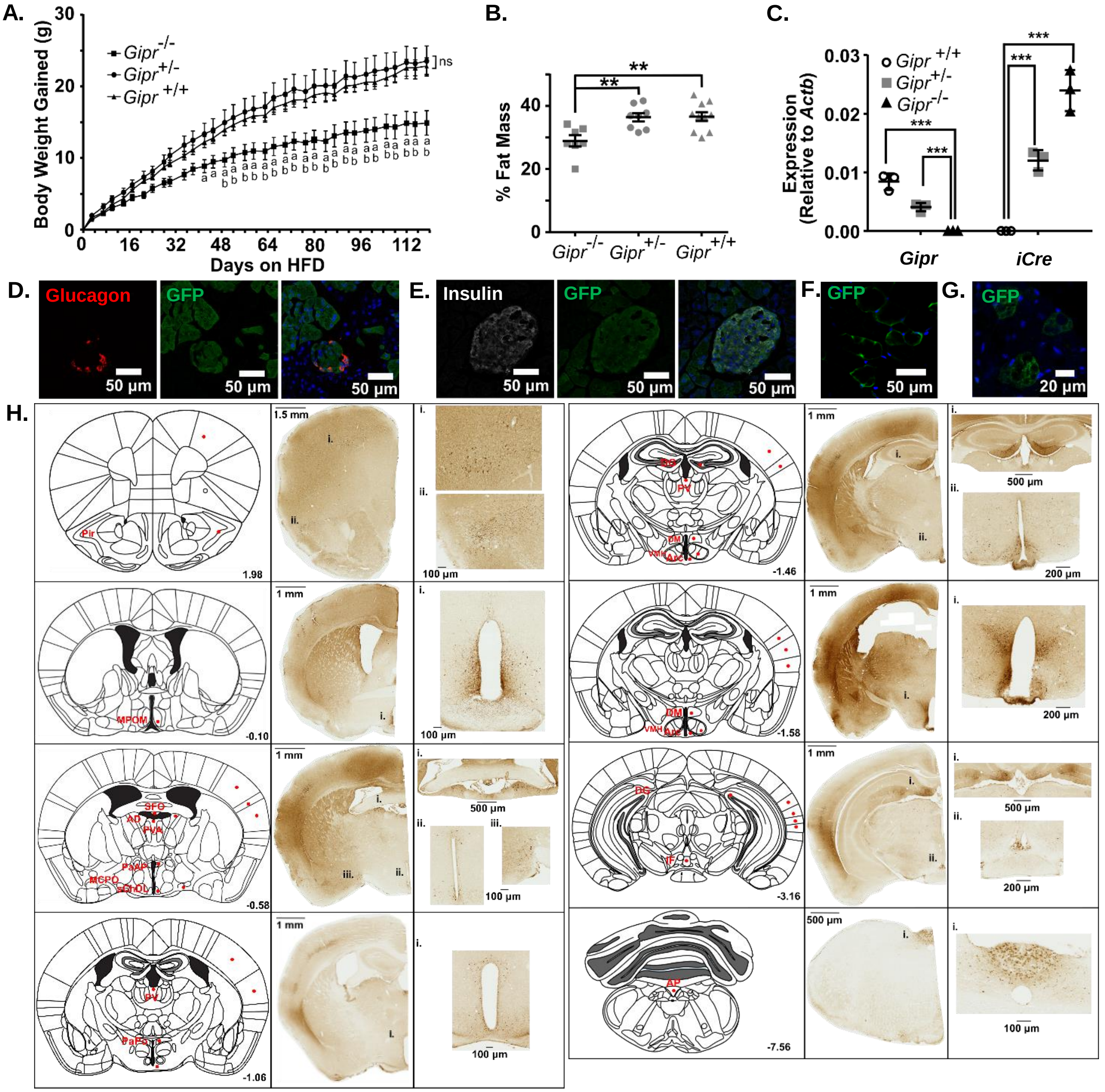


**Figure S1: *Gipr*-Cre mice allow for the manipulation and identification of *Gipr*-expressing cells *in vivo*** (Related to Figure 1)**:**

A. *Gipr*-Cre mice heterozygous for Cre at the *Gipr* locus were crossed producing offspring null, heterozygous, or homozygous for *Gipr*. Body weight was measured in response to 17 weeks on high fat diet. Significance in weight gain was tested via 2-way ANOVA with a Tukey's post hoc test; 'a' indicates P < 0.05 for *Gipr* $^{-/-}$ vs *Gipr* $^{+/-}$, 'b' indicates P < 0.05 *Gipr* $^{-/-}$ vs *Gipr* $^{+/+}$; n = 7 (*Gipr* $^{-/-}$), 8 (*Gipr* $^{+/-}$), 10 (*Gipr* $^{+/+}$). B. After 17 weeks on HFD, body composition was measured via NMR. Significance was determined using one-way ANOVA with a Tukey's post hoc test; n = 7 (*Gipr* $^{-/-}$), 8 (*Gipr* $^{+/-}$), 10 (*Gipr* $^{+/+}$). C. Relative expression of *iCre* and *Gipr* in whole islets isolated from *Gipr* $^{+/+}$, *Gipr* $^{+/-}$, *Gipr* $^{-/-}$ mice (n=3 for each genotype). Data are plotted as $2^{\Delta Ct}$ compared to *Actb* with bars representing mean +/- SD. Statistical significance was assessed through one-way ANOVA. Pancreas tissue from *Gipr*$^{EYFP}$ mice was stained for GFP (green) and glucagon (red, D), or insulin (white, E). Inguinal white (F) and interscapular brown (G) adipose tissue from *Gipr*$^{EYFP}$ mice was stained for GFP (green). H. Coronal sections from heterozygous *Gipr*$^{EYFP}$ and heterozygous *Gipr*$^{GCaMP3}$ mice were stained for GFP. Red circles represent the presence of GFP-immunoreactive somata. Drawings are based on the Paxinos Mouse Brain Atlas with the numerical values in the bottom right corner indicating the A/P location relative to Bregma. Pir, piriform cortex; MPOM, medial preoptic nucleus; SFO, subfornical organ; AD, anterodorsal thalamic nucleus; PVA/PV, paraventricular thalamic nucleus; PaAP/PaPo, paraventricular hypothalamic nucleus; MCPO, magnocellular preoptic nucleus; sChDL, suprachiasmatic nucleus; DG, dentate gyrus; ARC, arcuate nucleus; DM, dorsomedial hypothalamus; IF, interfasicular nucleus; AP, area postrema.

A.

| | *Snap25* | *Sst* | *Avp* | *Pthlh* | *Slc32a1* | *Cartpt* | *Cnr1* | *Tac1* | *Slc17a6* | *Nts* | *Pomc* | *Htr2c* | *Unc13c* | *Cckbr* | *Ghsr* | *Th* | *Lepr* | *Npy* | *Mc4r* | *Agrp* |
|---|---|---|---|---|---|---|---|---|---|---|---|---|---|---|---|---|---|---|---|---|
| *Snap25* | 229 | 189 | 133 | 114 | 100 | 97 | 89 | 75 | 63 | 20 | 19 | 9 | 8 | 8 | 3 | 3 | 3 | 2 | 2 | 0 |
| *Sst* | 189 | 197 | 123 | 105 | 94 | 97 | 83 | 62 | 48 | 17 | 18 | 5 | 9 | 8 | 3 | 3 | 3 | 2 | 1 | 0 |
| *Avp* | 133 | 123 | 141 | 68 | 70 | 63 | 62 | 49 | 40 | 13 | 14 | 5 | 9 | 7 | 3 | 2 | 3 | 2 | 1 | 0 |
| *Pthlh* | 114 | 105 | 68 | 117 | 77 | 69 | 56 | 40 | 19 | 11 | 10 | 1 | 3 | 2 | 0 | 3 | 1 | 0 | 0 | 0 |
| *Slc32a1* | 100 | 94 | 70 | 77 | 101 | 69 | 43 | 27 | 7 | 14 | 10 | 0 | 3 | 1 | 2 | 3 | 1 | 0 | 0 | 0 |
| *Cartpt* | 97 | 97 | 63 | 69 | 69 | 100 | 41 | 34 | 18 | 12 | 11 | 2 | 7 | 3 | 0 | 3 | 1 | 2 | 0 | 0 |
| *Cnr1* | 89 | 83 | 62 | 56 | 43 | 41 | 95 | 51 | 42 | 9 | 7 | 4 | 1 | 4 | 1 | 3 | 2 | 0 | 0 | 0 |
| *Tac1* | 75 | 62 | 49 | 40 | 27 | 34 | 51 | 80 | 45 | 6 | 7 | 8 | 4 | 4 | 0 | 1 | 1 | 1 | 1 | 0 |
| *Slc17a6* | 63 | 48 | 40 | 19 | 7 | 18 | 42 | 45 | 68 | 7 | 7 | 8 | 5 | 6 | 1 | 0 | 1 | 2 | 1 | 0 |
| *Nts* | 20 | 17 | 13 | 11 | 14 | 12 | 9 | 6 | 7 | 20 | 3 | 1 | 1 | 1 | 0 | 1 | 0 | 1 | 0 | 0 |
| *Pomc* | 19 | 18 | 14 | 10 | 10 | 11 | 7 | 7 | 7 | 3 | 19 | 0 | 3 | 2 | 1 | 1 | 0 | 1 | 0 | 0 |
| *Htr2c* | 9 | 5 | 5 | 1 | 0 | 2 | 4 | 8 | 8 | 1 | 0 | 9 | 1 | 0 | 0 | 0 | 1 | 0 | 1 | 0 |
| *Unc13c* | 8 | 9 | 9 | 3 | 3 | 7 | 1 | 4 | 5 | 1 | 3 | 1 | 9 | 2 | 0 | 0 | 0 | 2 | 0 | 0 |
| *Cckbr* | 8 | 8 | 7 | 2 | 1 | 3 | 4 | 4 | 6 | 1 | 2 | 0 | 2 | 8 | 0 | 0 | 0 | 2 | 0 | 0 |
| *Ghsr* | 3 | 3 | 3 | 0 | 2 | 0 | 1 | 0 | 1 | 0 | 1 | 0 | 0 | 0 | 3 | 0 | 0 | 0 | 0 | 0 |
| *Th* | 3 | 3 | 2 | 3 | 3 | 3 | 3 | 1 | 0 | 1 | 1 | 0 | 0 | 0 | 0 | 3 | 0 | 0 | 0 | 0 |
| *Lepr* | 3 | 3 | 3 | 1 | 1 | 1 | 2 | 1 | 1 | 0 | 0 | 1 | 0 | 0 | 0 | 0 | 3 | 0 | 1 | 0 |
| *Npy* | 2 | 2 | 2 | 0 | 0 | 2 | 0 | 1 | 2 | 1 | 1 | 0 | 2 | 2 | 0 | 0 | 0 | 2 | 0 | 0 |
| *Mc4r* | 2 | 1 | 1 | 0 | 0 | 0 | 0 | 1 | 1 | 0 | 0 | 1 | 0 | 0 | 0 | 0 | 1 | 0 | 2 | 0 |
| *Agrp* | 0 | 0 | 0 | 0 | 0 | 0 | 0 | 0 | 0 | 0 | 0 | 0 | 0 | 0 | 0 | 0 | 0 | 0 | 0 | 0 |

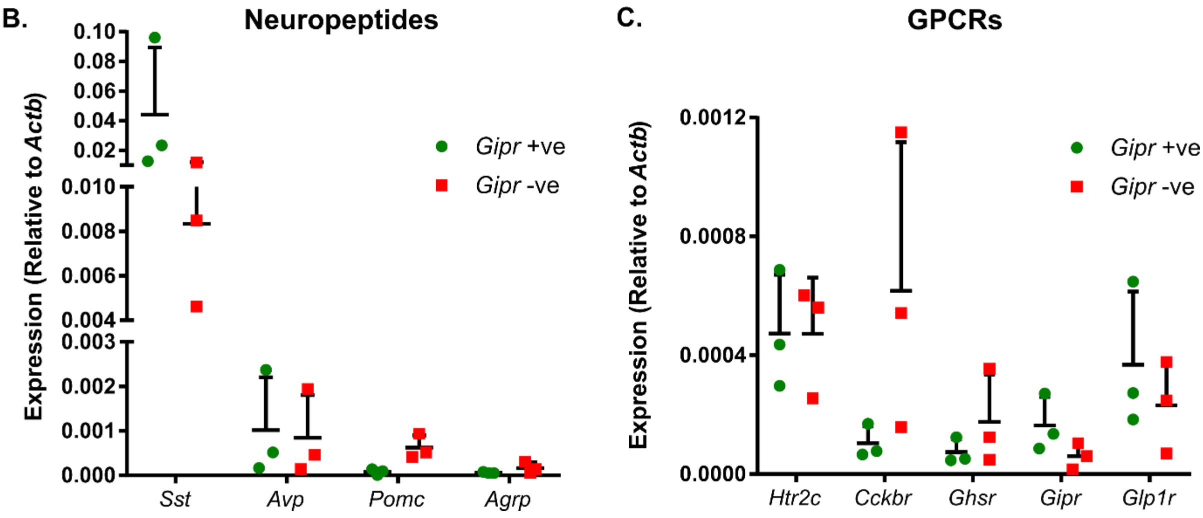


**Figure S2. Expression of selected neuroendocrine targets in *Gipr* cells** (Related to Figure 1)**:**
A. Expression matrix showing the number of cells from the neuronal cluster expressing a selection of neuroendocrine genes. RNA was extracted from FACS purified hypothalamic *Gipr*-expressing cells from heterozygous *Gipr*$^{EYFP}$ or *Gipr*$^{GCaMP3}$ mice and converted to cDNA. Gene expression of selected neuropeptides (B) and cell surface receptors (C) in *Gipr*-positive and *Gipr*-negative cells was measured by qPCR. Data are plotted as $2^{\Delta Ct}$ compared to *Actb* with bar representing mean + SD. n = 3 sorts, equivalent to 8 mice.

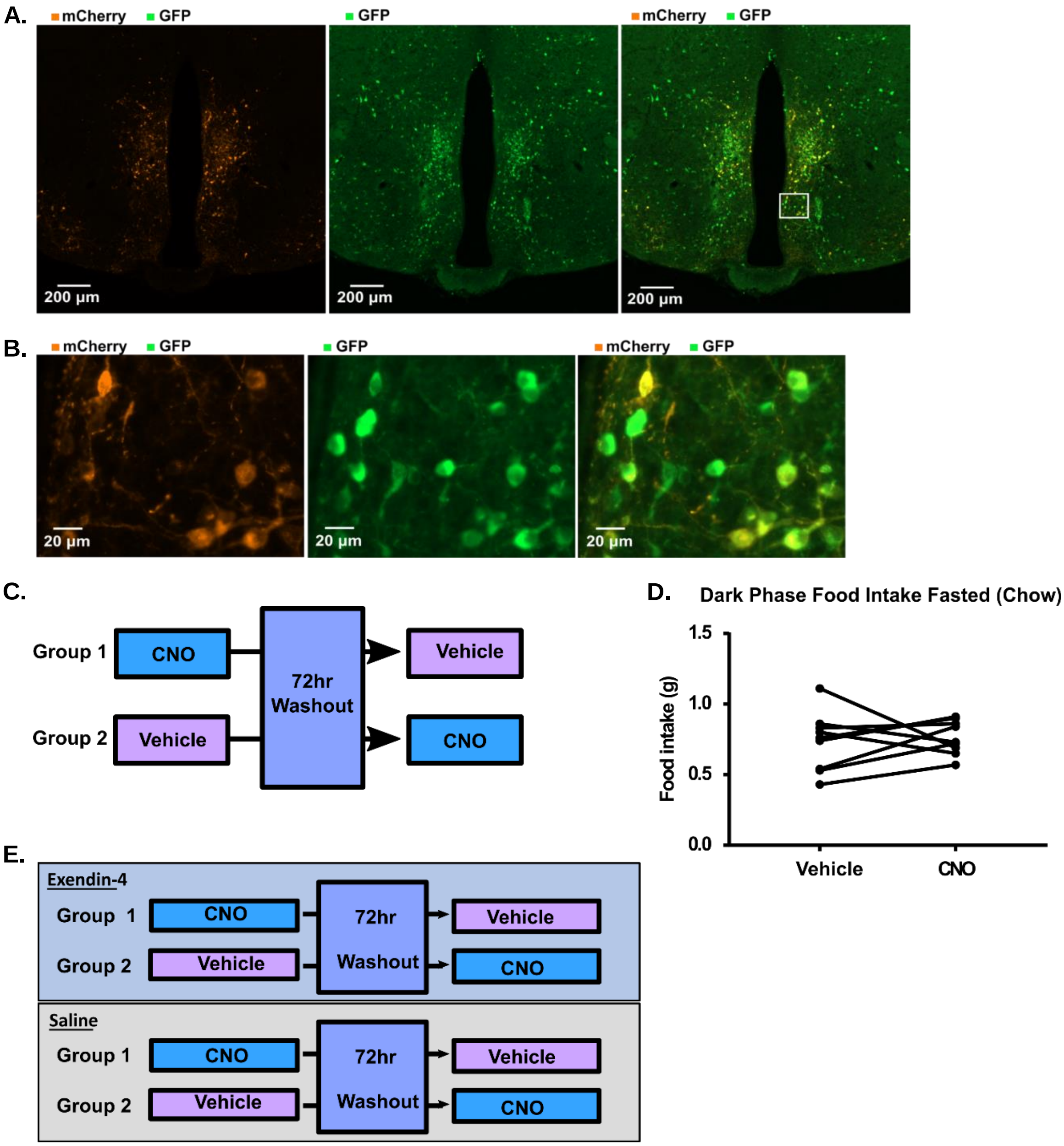


**Figure S3: Targeting of AAV-DIO-hM3D-mCherry to *Gipr* cells; Food intake study design** (Related to Figure 3,4)**:**

Brains were harvested from *Gipr*[hypDq] mice. Coronal sections were stained for GFP and mCherry to assess the targeting of hM3D-mCherry into *Gipr*-expressing cells. A. Representative slice showing GFP (green) and mCherry (red) staining. B. Enlarged image of cells demarcated by white box in panel A. C. Illustration of crossover design used for experiments represented in Figure 3 and 4F. Each mouse served as its own control. D. Heterozygous *Gipr*-Cre mice that had not received injections of AAV-DIO-hM3D-mCherry were administered either CNO (1 mg/kg) or vehicle i.p following a 10hr daytime fast in a crossover design study. The amount of food consumed during the beginning of the dark phase was measured 2hr post-injection and compared using a paired t-test. n= 9. E. Illustration of crossover study design used for experiments represented in Figure 4G. Each mouse served as its own control.

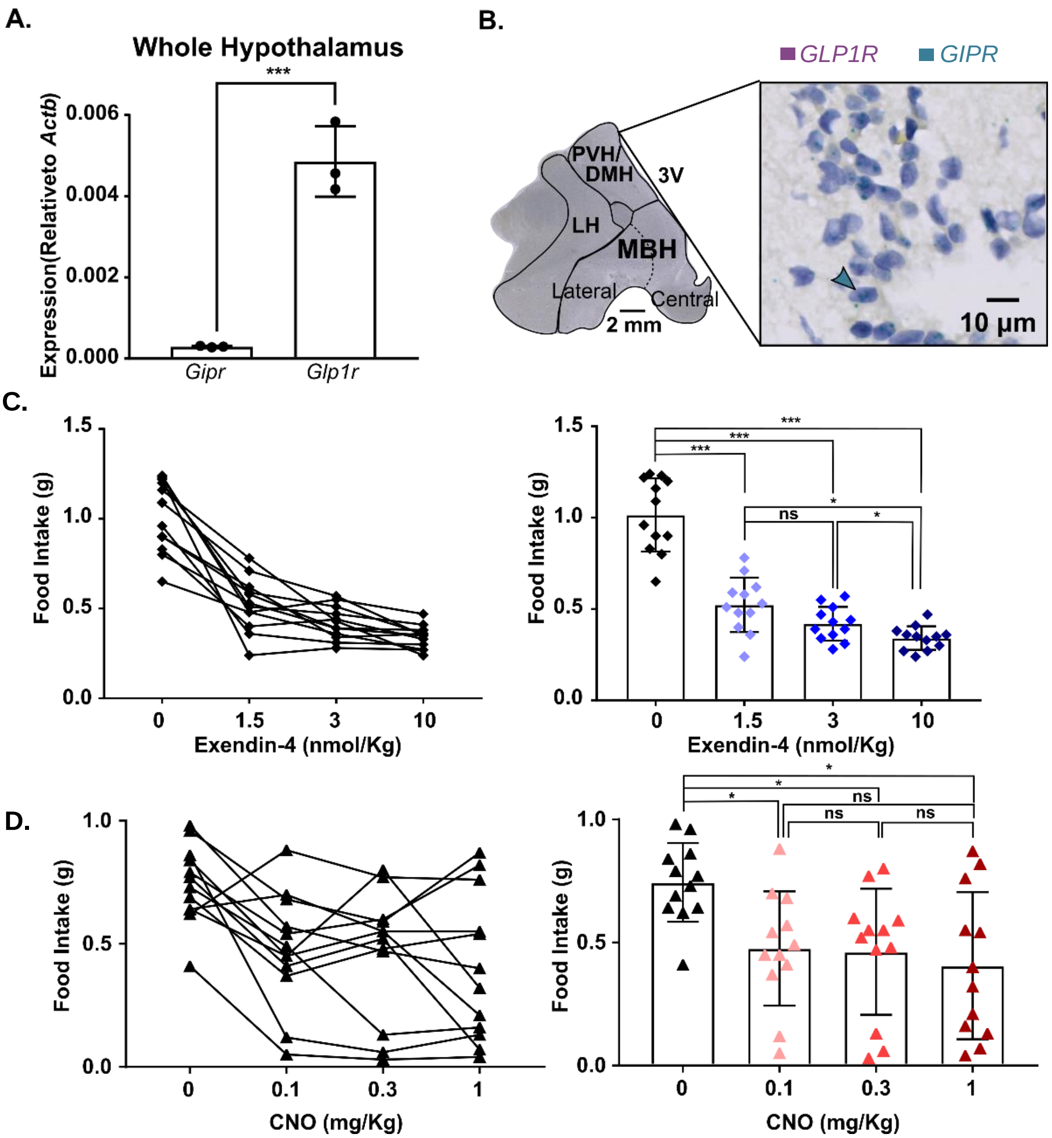


**Figure S4: Hypothalamic *Gipr* localisation; CNO/Ex-4 dose response** (Related to Figure 4)**:**
A. Relative expression of *Gipr* and *Glp1r* in whole hypothalamic homogenates in WT mice (n=3). Data are plotted as $2^{\Delta Ct}$ compared to *Actb* with bars representing mean +/- SD. Expression levels of *Gipr* and *Glp1r* were compared using an unpaired student's t test. ***P<0.001; n =3. B. Human hypothalamus tissue samples were labelled for *GIPR* and *GLP1R* mRNA using RNAscope. Cells lining the third ventricle (3V), likely to be ependymal cells expressing *GIPR*. C. Following a 10 h daytime fast heterozygous *Gipr*-Cre and *Gipr* WT mice were injected with Ex-4 (1.5, 3, or 10 nmol/kg) or saline s.c. 1hr prior to the onset of the dark phase. At the onset of the dark phase food was presented, and food intake measurements were taken 2hr later. This was a crossover design study where each mouse served as its own control. Food intake was compared using a repeated measures 2-way ANOVA with a Sidak's post hoc test. *P<0.05, ***P<0.001; n = 12. D. Following a 10hr daytime fast *Gipr*$^{\text{hypDq}}$ mice were injected with saline s.c. 1hr prior to the onset of the dark phase. CNO (0.1, 0.3, or 1 mg/kg) or vehicle was injected i.p. at the onset of the dark phase, food was presented, and food intake measurements were taken 2hr post-activation. This was a crossover design study where each mouse served as its own control. Food intake was compared using a repeated measures 2-way ANOVA with a Sidak's post hoc test. *P<0.05; n = 12.
