# Supplementary material for: Glucose-Dependent Insulinotropic Polypeptide Receptor-Expressing Cells in the Hypothalamus Regulate Food Intake
Source: Cell Metab. 2019 Nov 5;30(5):987–996.e6. doi: 10.1016/j.cmet.2019.07.013 (PMC6838660; doi:10.1016/j.cmet.2019.07.013)
Supplement: Document S2. Article plus Supplemental Information [file mmc3.pdf]

Short Article

Cell Metabolism

Glucose-Dependent Insulinotropic Polypeptide Receptor-Expressing Cells in the Hypothalamus Regulate Food Intake

Graphical Abstract

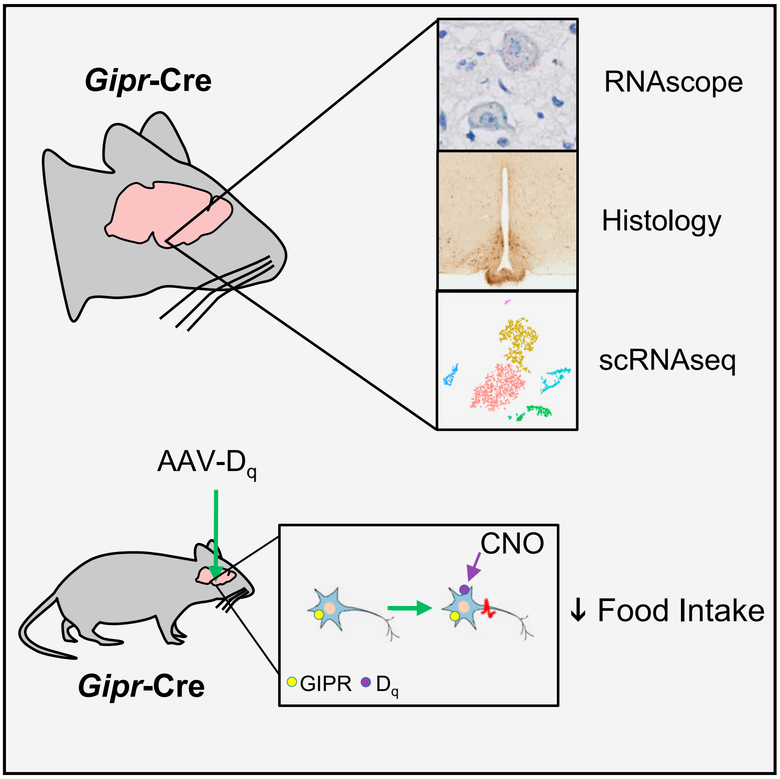

Highlights

- *Gipr* is expressed in the hypothalamus, as demonstrated using a new *Gipr*-Cre mouse model
- *Gipr* cells included somatostatin-positive neurons, glia, and vascular cells
- *Gipr* overlapped partially with *Glp1r* in human and mouse hypothalamus by RNAscope
- Activation of *Gipr* neurons using local AAV-delivered G_q-DREADDs reduced food intake

Authors

Alice E. Adriaenssens, Emma K. Biggs, Tamana Darwish, ..., Clemence Blouet, Fiona M. Gribble, Frank Reimann

Correspondence

fmg23@cam.ac.uk (F.M.G.), fr222@cam.ac.uk (F.R.)

In Brief

Adriaenssens et al. identified cells expressing receptors for the gut hormone GIP in human and mouse hypothalamus using a new *Gipr*-Cre mouse model and RNAscope. Local hypothalamic delivery of AAVs expressing Cre-dependent G_q-DREADDs revealed that activation of *Gipr*-positive neurons in mice reduced food consumption.

Adriaenssens et al., 2019, Cell Metabolism *30*, 987–996
November 5, 2019 © 2019 The Authors. Published by Elsevier Inc.
https://doi.org/10.1016/j.cmet.2019.07.013

Cell Metabolism
Short Article

Glucose-Dependent Insulinotropic Polypeptide Receptor-Expressing Cells in the Hypothalamus Regulate Food Intake

Alice E. Adriaenssens,[1,2] Emma K. Biggs,[1,2] Tamana Darwish,[1] John Tadross,[1] Tanmay Sukthankar,[1] Milind Girish,[1] Joseph Polex-Wolf,[1] Brain Y. Lam,[1] Ilona Zvetkova,[1] Warren Pan,[1] Davide Chiarugi,[1] Giles S.H. Yeo,[1] Clemence Blouet,[1] Fiona M. Gribble,[1,*] and Frank Reimann[1,3,*]
[1]Metabolic Research Laboratories, Wellcome Trust MRC Institute of Metabolic Science, Addenbrooke's Hospital, Hills Road, Cambridge CB2 0QQ, UK
[2]Senior author
[3]Lead Contact
*Correspondence: fmg23@cam.ac.uk (F.M.G.), fr222@cam.ac.uk (F.R.)
https://doi.org/10.1016/j.cmet.2019.07.013

SUMMARY

Ambiguity regarding the role of glucose-dependent insulinotropic polypeptide (GIP) in obesity arises from conflicting reports asserting that both GIP receptor (GIPR) agonism and antagonism are effective strategies for inhibiting weight gain. To enable identification and manipulation of *Gipr*-expressing (*Gipr*) cells, we created *Gipr*-Cre knockin mice. As GIPR-agonists have recently been reported to suppress food intake, we aimed to identify central mediators of this effect. *Gipr* cells were identified in the arcuate, dorsomedial, and paraventricular nuclei of the hypothalamus, as confirmed by RNAscope in mouse and human. Single-cell RNA-seq identified clusters of hypothalamic *Gipr* cells exhibiting transcriptomic signatures for vascular, glial, and neuronal cells, the latter expressing somatostatin but little pro-opiomelanocortin or agouti-related peptide. Activation of G_q-DREADDs in hypothalamic *Gipr* cells suppressed food intake *in vivo*, which was not obviously additive with concomitant GLP1R activation. These data identify hypothalamic GIPR as a target for the regulation of energy balance.

INTRODUCTION

Glucose-dependent insulinotropic polypeptide (GIP) is a gut hormone released from enteroendocrine cells in the duodenum and jejunum (Buchan et al., 1978; Buffa et al., 1975) within minutes of ingesting a meal (Elliott et al., 1993). GIP binds its cognate receptor, GIP receptor (GIPR)—a class B G-protein-coupled receptor (GPCR) (Usdin et al., 1993). GIPR activation stimulates insulin release in pancreatic beta cells (Dupre et al., 1973), and together with its sister incretin, glucagon-like peptide-1 (GLP-1), GIP is an important glucostat to keep post-prandial blood glucose levels in check. It is GLP-1, however that has enjoyed the therapeutic limelight in efforts to design more effective type 2 diabetes treatments. Initial interest in GIP-based therapies waned following studies showing that the insulinotropic properties of GIP are attenuated in patients with type 2 diabetes (Nauck et al., 1993). The complex relationship between GIP and adiposity has further obscured our understanding of GIP's therapeutic potential.

Studies showing that genetic or pharmacological blockade of GIPR protects against obesity have implicated GIP in promoting body weight gain (McClean et al., 2007; Miyawaki et al., 2002). These data are congruent with GIP's role in facilitating triglyceride storage in adipose tissue (Eckel et al., 1979; Wasada et al., 1981). Though GIPR antagonists have inhibited weight gain in animal models (Boylan et al., 2015; Fulurija et al., 2008; Killion et al., 2018; McClean et al., 2007), the therapeutic utility of GIPR antagonism in humans is yet to be determined. The recent realization that some peptides designed to be GIPR antagonists exhibit partial agonist activity (Sparre-Ulrich et al., 2015), confounding the interpretation of some of these studies, as well as evidence indicating that the lipogenic action of GIP may be mediated indirectly via insulin (Campbell et al., 2016; Ugleholdt et al., 2011), has led some to question the rationale behind blocking

Context and Significance

Several drugs against diabetes and obesity are based on the gut hormone glucagon-like peptide-1 (GLP-1), which controls appetite and blood sugar levels. The related gut hormone glucose-dependent insulinotropic polypeptide (GIP) also controls blood glucose, but its role in food intake is debated. Researchers at the Institute of Metabolic Science in Cambridge, UK identified neurons expressing the GIP receptor in food intake control centers of mouse and human brain. Activating these neurons in mice reduced feeding, providing a neuronal circuit that could underlie the results of recent clinical trials of GLP-1-GIP dual agonist peptides, which suggested that GIP may work together with GLP-1 to suppress appetite. Understanding the role of this neuronal population responsive to GIP will help the development of new drugs targeting diabetes and obesity.

© 2019 The Authors. Published by Elsevier Inc.
This is an open access article under the CC BY license (http://creativecommons.org/licenses/by/4.0/).

GIP signaling as a route toward tackling obesity (Finan et al., 2016).

Augmenting GIPR signaling in combination with proven antidiabetic agents has yielded exciting results. In rodents and humans, GLP-1-GIP dual agonism significantly improved glycemic control and provided greater body weight loss compared to treatment with a GLP-1 receptor agonist alone (Coskun et al., 2018; Finan et al., 2013; Frias et al., 2018; Nørregaard et al., 2018). In mice, this additional weight loss could be attributable to a further reduction in food intake (Coskun et al., 2018; Finan et al., 2013; Nørregaard et al., 2018). It is tempting to suggest that the addition of GIPR activation underlies the superior performance of these combinatorial therapies (Coskun et al., 2018; DiMarchi, 2018), although GIPR-only agonists appear to either not or fairly modestly reduce body weight when given in isolation (Coskun et al., 2018; Mroz et al., 2019). While GLP-1 exhibits central inhibitory actions on food intake (Turton et al., 1996), comparatively little is known about the central activity of GIP on appetite or the expression profile of *Gipr* in the CNS. Previous attempts at visualizing the localization of *Gipr* in the CNS relied on traditional *in situ* hybridization or radiolabeled ligands (Kaplan and Vigna, 1994; Paratore et al., 2011; Usdin et al., 1993). While these studies did find evidence of *Gipr* in the cortex, hippocampus, and olfactory bulb, the low resolution of these methodologies does not allow for the precise mapping of *Gipr* production to distinct cells.

In this study, we sought to define the central GIP signaling axis and to understand how manipulation of *Gipr* cells in the brain affects feeding behavior. Through the use of a transgenic mouse, *Gipr*-Cre, we examined the location, transcriptomic profile, and effects of acute activation of *Gipr* cells in the CNS.

RESULTS

Gipr Is Expressed in Neurons and Glial Cells in Key Feeding Centers of the Brain

Although two GIPR antagonistic antibodies have been reported (Killion et al., 2018; Ravn et al., 2013), neither has been used for immunohistochemical localization. To label *Gipr* cells, we generated a knockin transgenic mouse model (*Gipr*-Cre) in which Cre-recombinase replaces the *Gipr* coding sequence, enabling the genetic and chemogenetic manipulation of *Gipr* cells; mice homozygous for *Gipr*-Cre are thus *Gipr* nulls. *Gipr* null offspring were protected against body weight gain when subjected to a high-fat diet (HFD) for 17 weeks and had significantly lower percent fat mass compared with *Gipr*-Cre heterozygous and wild-type (WT) littermates (Figures S1A and S1B), supporting previous results from another *Gipr* knock-out (KO) model (Miyawaki et al., 2002). Heterozygous *Gipr*-Cre mice, by contrast, gained a similar amount of weight as WT mice, despite reduced *Gipr* expression due to haploinsufficiency (Figure S1C). For the rest of this study, we used *Gipr*-Cre heterozygous animals.

By crossing *Gipr*-Cre mice with *ROSA26*-EYFP reporter mice (*Gipr*^{EYFP}), we identified *Gipr* cells in target tissues. Staining for EYFP in the pancreas of *Gipr*^{EYFP} mice reported expression of *Gipr* in both alpha and beta cells, as expected. Heterogeneous EYFP staining was also found in the surrounding pancreatic exocrine tissue (Figures S1D and S1E). A proportion of adipocytes in interscapular brown and inguinal white adipose tissue stained positively for EYFP (Figures S1F and S1G). These data provided confidence that the *Gipr*-Cre transgene successfully targets *Gipr* expressing cells, as they are consistent with known expression patterns for *Gipr* (Campbell and Drucker, 2013).

To create a map of central *Gipr* localization, brains of *Gipr*^{EYFP} mice were serially sectioned and stained for EYFP. In line with *in situ* and radioligand binding data (Kaplan and Vigna, 1994; Paratore et al., 2011; Usdin et al., 1993), staining was fairly widespread within the CNS (Figure S1H), including key feeding centers of the hypothalamus, such as the arcuate (ARC), paraventricular (PVN), and dorsomedial hypothalamic (DMH) nuclei (Figure 1A). Active transcription of *Gipr* in the adult hypothalamus was confirmed by qPCR (Figure 1B).

To create a transcriptomic profile of *Gipr* cells in the hypothalamus, cell preparations from the hypothalami of *Gipr*^{EYFP} mice were purified using fluorescence-activated cell sorting (FACS), and their transcriptomes were analyzed via single-cell RNA sequencing (scRNA-seq). Graph-based clustering analysis revealed that hypothalamic *Gipr* cells separate into six subpopulations (Figure 1C top). Cluster identities were assigned based on the expression patterns of cell-type-specific genes, including those found in the most enriched cluster markers (Figures 1C [bottom] and 1D, and Table S1), with mural cells (*Kcnj8*, *Abcc9*, *Mustn1*, and *Mhy11*), ependymocytes (*Ccdc153* and *Hdc*), vascular and leptomeningeal cells (VLMC) (*Lum* and *Pdgfra*), oligodendrocytes (*Mal* and *Klk6*), and neurons (*Snap25* and *Syt1*) representing distinct clusters of *Gipr* cells.

As hypothalamic neurons are known to modulate feeding behavior, we analyzed the neuronal cluster in more detail. *Gipr* neurons expressed markers for both GABAergic (*Slc32a1*) and glutamatergic (*Slc17a6*) cells (Figure 1D). Genes encoding peptides previously implicated in energy homeostasis, namely *Sst*, *Avp*, *Tac1*, and *Cartpt*, were among the most highly expressed neurohormonal markers (Figure 1E). To examine the heterogeneity of *Gipr*-fluorescent neurons, we constructed a matrix showing the numbers of individual *Gipr* cells from the neuronal cluster co-expressing a selection of 20 genes implicated in neuroendocrine signaling pathways (Figure S2A). *Sst* was the primary neuroendocrine marker for *Gipr* neurons with 83% of *Snap25*-positive cells in the neuronal cluster expressing *Sst*. *Avp* and *Pthlh* were also expressed in at least half of the *Gipr* neurons (58% and 50%), with *Cartpt* and *Tac1* expressed in fewer than 50%. *Pomc* was expressed in less than 10% of *Gipr* neurons and only at low levels. Consistent with these scRNA-seq results, we observed an apparent enrichment in *Sst* and diminished *Pomc* message by qRT-PCR in independently isolated fluorescently labeled *Gipr* cells (Figure S2B).

Local and Peripheral Signals Regulate *Gipr* Neurons

To identify regulatory cell surface receptors present in *Gipr* neurons, we analyzed the expression of GPCRs in the neuronal cluster. *Grm5* and *Gabbr1* were the most highly expressed GPCRs in *Gipr* neurons, which also expressed ionotropic receptors for glutamate and GABA (*Gria2*, *Gria3*, *Grin2b*, and *Gabrb1*; data not shown). Other neurotransmitters likely to contribute to *Gipr* neuron regulation include opioids (via *Oprk1* and *Oprl1*), acetylcholine (via *Chrm1* and *Chrm3*), histamine (*Hrh3*), and serotonin (*Htr1b*, *Htr1d*, and *Htr2c*). *Gipr* neurons also expressed

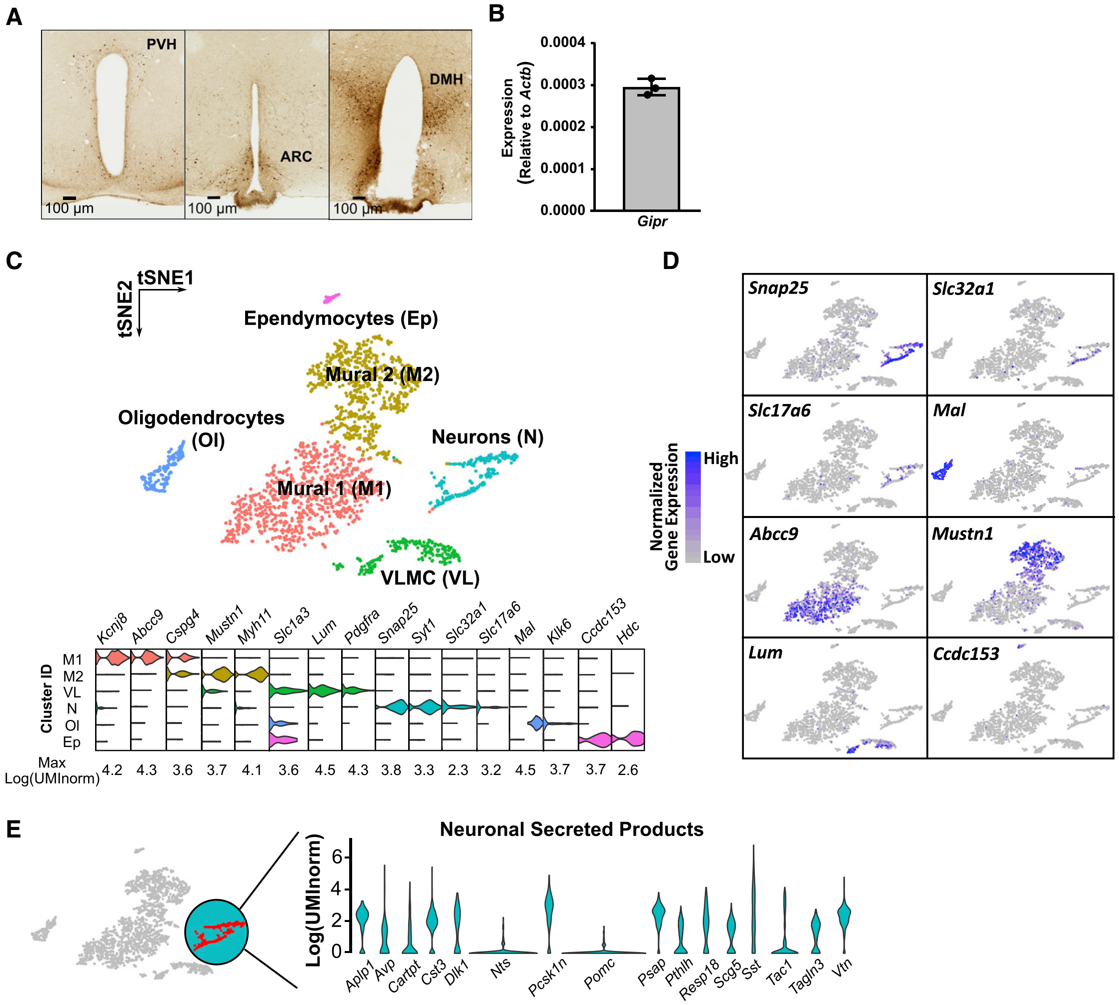

Figure 1. *Gipr*-Expressing Cells in the Brain

(A) Micrograph of GFP staining in brain from heterozygous *Gipr*^{EYFP} mice (see also Figure S1).

(B) Relative expression of *Gipr* in whole hypothalamic homogenates in WT mice (n = 3). Data are plotted as $2^{\Delta Ct}$ compared to *Actb* with the bar representing mean ± SD.

(C) *Gipr* cells were isolated from single-cell digests of hypothalami from two heterozygous *Gipr*^{EYFP} mice via FACS, and their transcriptomes were analyzed by scRNA-seq followed by clustering analysis. tSNE visualization of hypothalamic *Gipr* cells indicates that there are six clusters (top). Cell types were assigned according to expression of a combination of marker genes (bottom) (see also Table S1).

(D) t-SNE plots of the expression of selected markers for neurons (*Snap25*), GABAergic neurons (*Slc32a1*), glutamatergic neurons (*Slc17a6*), oligodendrocytes (*Mal*), mural cells (*Abcc9* and *Mustn1*), VLMCs (*Lum*), and ependymocytes (*Ccdc153*).

(E) Violin plots representing expression of genes encoding secreted products within the neuronal cluster.

receptors for peptide neuroendocrine regulators, including SST (*Sstr2* and *Sstr1*), calcitonin (*Calcr*), and PACAP (*Calcrl*) and expressed receptors known to govern energy balance, including *Cnr1*, *Mchr1*, *Hcrtr2*, *Tac1r*, *Ghsr*, *Cckbr*, and *Htr2c* (Figure 2A).

The functional activity of several receptors was interrogated at the single-cell level using calcium imaging in cultured hypothalamic neurons from *Gipr*^{GCaMP3} mice. The receptors selected were *Cckbr*, *Ghsr*, and *Htr2c*, the expression of which we confirmed by qRT-PCR in additional hypothalamic FACS sorts (Figure S2C). Given the finding that some *Gipr* neurons expressed *Cartpt* and *Pomc*, we also examined *Lepr*, which has previously been shown to induce calcium influx in POMC neurons (Heeley et al., 2018; Smith et al., 2018). Of the stimuli tested, glutamate increased calcium in the majority (40/59) of neurons, while only subsets responded to CCK (5/34) and the GHSR agonist hexarelin (7/73). Only 1/15 neurons responded to leptin and no cells responded to 5HT (Figures 2B and 2C).

Activation of Hypothalamic *Gipr* Cells Decreases Food Intake

To assess the effect of acute chemogenetic manipulation of *Gipr* cell activity on food intake, *Gipr*-Cre mice received hypothalamic injections of Cre-inducible AAVs expressing the G_q-coupled DREADD, hM3D (AAV-*hSyn*-DIO-hM3D(G_q)-mCherry) (Armbruster et al., 2007), designed to preferentially target neurons (Hammond et al., 2017; Kügler et al., 2003), to produce *Gipr*^{hypDq}

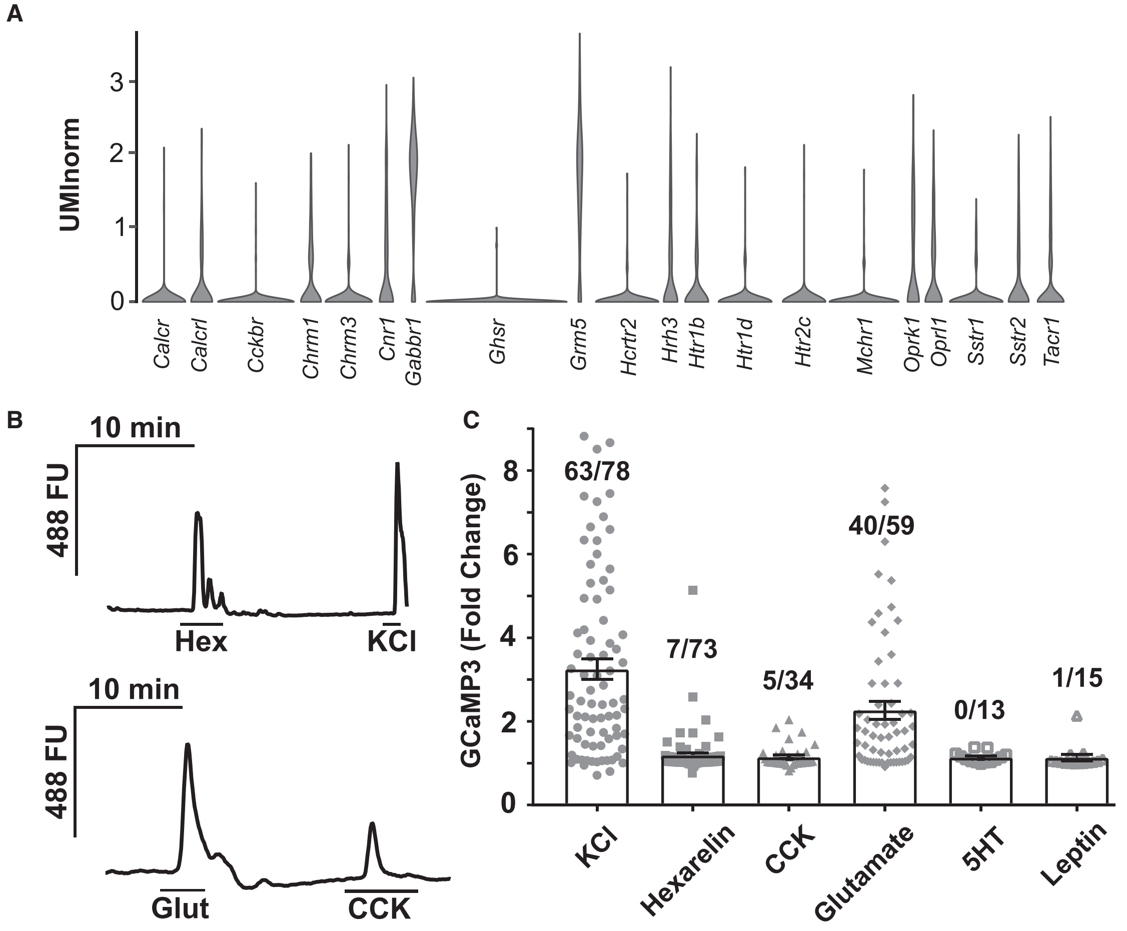

Figure 2. *Gipr*-Expressing Cells Are Activated by Endocrine Factors

(A) Violin plots depicting the expression of GPCRs in cells from the neuronal cluster.

(B and C) Ligands for a selection of receptors were tested using calcium imaging in primary cultures of adult hypothalamic cells from heterozygous *Gipr*^{GCaMP3} mice. Dispersed hypothalamic cells were imaged 2–16 h after plating. Cells were perfused with stimuli as indicated. Example traces are shown in (B), and data from all cells tested are represented in (C), with the number of responding cells out of the total number imaged for each condition represented above each bar. Bars represent the mean ± SE.

mice (Figures S3A and S3B). Food intake effects of *Gipr*-cell D_q activation were assessed in a crossover study (Figure S3C).

In chow-fed *Gipr*^{hypDq} mice, activation of D_q receptors following injection of clozapine-N-oxide (CNO) significantly suppressed both light- and dark-phase food intake in *ad lib*-fed and fasted animals (Figures 3A–3C). Similarly, CNO injected at the onset of the dark phase in *Gipr*^{hypDq} mice fed an HFD for 2–4 weeks significantly reduced food intake, but no significant effect was observed when CNO was injected at the start of the light phase (Figures 3D–3F). No effect on food intake was observed in control (non-AAV-injected) mice following administration of CNO (Figure S3D).

Gipr and *Glp1r* Are Co-expressed in a Subset of Cells in Humans and Mice

To investigate potential overlap between *Gipr* and GLP-1 receptor (*Glp1r*) expression, we performed RNAscope analysis of mouse and human hypothalamus. Consistent with the cellular localization identified using *Gipr*^{EYFP} mice, RNAscope revealed *Gipr*-positive cells in mouse ARC and DMH. *Glp1r*-positive cells were also observed in these nuclei, with some cells exhibiting both *Gipr* and *Glp1r* expression (Figures 4A–4C). In human hypothalamic sections, we similarly observed cells positive for *GIPR*, *GLP1R*, or both receptors together (Figures 4D and 4E). It was noticeable that in both mouse and human, the probes for *Glp1r* or *GLP1R* detected a higher density of transcripts per cell than the *Gipr* or *GIPR* probes. In mice, this is in accordance with lower expression of *Gipr* measured by qPCR compared to *Glp1r* in FACS-purified hypothalamic *Gipr* cells (Figure S2C) as well as homogenates of whole hypothalamus (Figure S4A). In human, *GIPR* signal was also observed in periventricular cells in the ependymal region (Figure S4B).

Co-activation of *Gipr* and *Glp1r* Cells Does Not Further Reduce Acute Food Intake

To investigate potential additive effects of simultaneously activating hypothalamic *Gipr*- and *Glp1r*-positive cells on acute food intake, we first injected AAVs carrying *hSyn*-DIO-hM3D(G_q)-mCherry into the hypothalamus of mice expressing Cre under the control of both the *Gipr* and *Glp1r* promoters

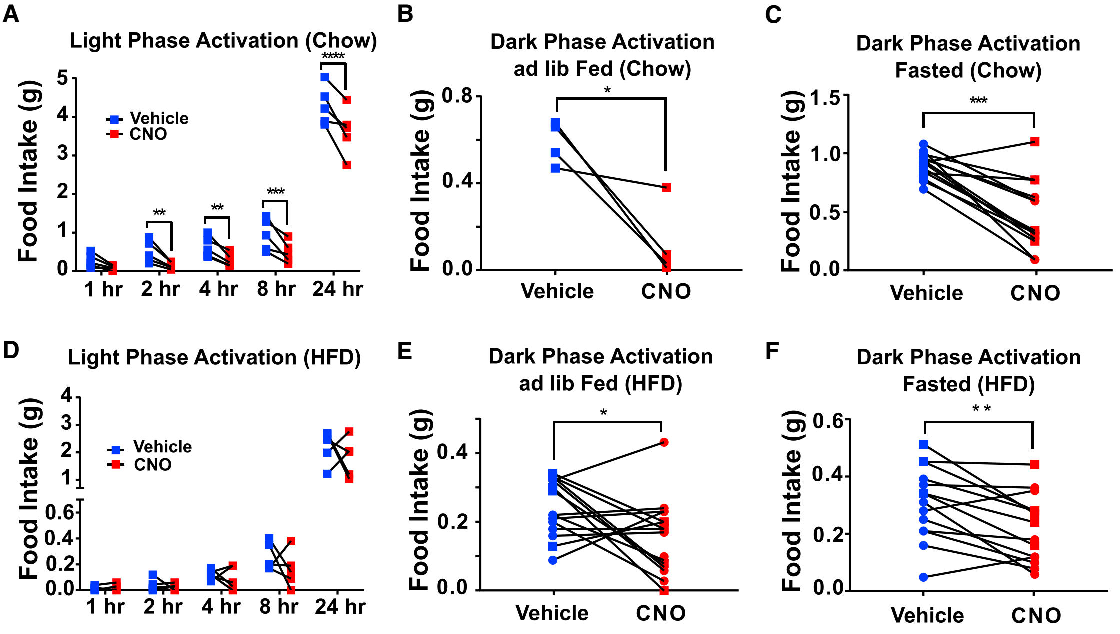

Figure 3. Activation of Hypothalamic *Gipr*-Expressing Cells Decreases Food Intake

Heterozygous *Gipr*-Cre mice were injected bilaterally with AAV-DIO-hM3D-mCherry into the hypothalamus to produce *Gipr*^{hypDq} mice. CNO (1 mg/kg) or vehicle was injected i.p. following either *ad lib* feeding or a 10-h daytime fast before dark-phase food intake or following a 2-h fast for light-phase measurements. These paradigms were tested in both chow- (A)–(C) and HFD- (D)–(F) fed mice. Different symbols (squares and circles) indicate mice from different experimental cohorts (see also Figure S3). Dark-phase food intake was compared using a paired t test. Light-phase food intake was compared using a repeated measures 2-way ANOVA with a Sidak's post-hoc test. *$p < 0.05$, **$p < 0.01$, ***$p < 0.001$; n = 5 (A) and (D), 4 (B), 14 (C), 15 (E), and 14 (F).

(*Gipr/Glp1r*^{hypDq}) or the *Glp1r* promoter alone (*Glp1r*^{hypDq}). CNO injected at the start of the dark phase significantly reduced 2-h food intake in both fasted *Gipr/Glp1r*^{hypDq} and *Glp1r*^{hypDq} mice, although this did not reach statistical significance for the latter (Figure 4F). The CNO-dependent reduction in food intake of 52% ± 9% (n = 7) in *Gipr/Glp1r*^{hypDq} mice was, however, similar in magnitude to the previously observed 50% ± 8% (n = 14) reduction in *Gipr*^{hypDq} mice (Figure 3C).

In a second approach, we tested the effect of peripherally administered Exendin-4 (Ex-4) in combination with *Gipr* cell D_q activation on food intake. A sub-maximal dose of Ex-4 was chosen from dose-response trials (Figure S4C). While Ex-4, CNO, and the combination of Ex-4 with CNO all reduced 2-h food intake in *Gipr*^{hypDq} mice, we were unable to detect a significant difference between treatments (Figure 4G) on acute food intake.

DISCUSSION

In this study, we generated a new *Gipr*-Cre mouse model, identifying *Gipr*-expressing cells in the hypothalamus, and enabling their transcriptomic and functional characterization. We show that (1) *Gipr* is expressed in neuronal and non-neuronal cell types in key feeding centers of the brain, (2) hypothalamic *Gipr* neurons express a diverse range of neuroendocrine hormones and hormonal or neurotransmitter receptors, (3) direct activation of hypothalamic *Gipr* cells potently suppresses food intake, and (4) *Gipr* is co-expressed with *Glp1r* in a subset of hypothalamic cells in humans and mice.

Centrally expressed *Gipr* has previously been implicated in promoting neurogenesis and synaptic plasticity (Faivre et al., 2011; Nyberg et al., 2005; Paratore et al., 2011). Our finding that *Gipr* cells are present in the ARC, DMH, and PVH suggests that GIPR signaling may also integrate with well-characterized hypothalamic circuits regulating energy balance (Waterson and Horvath, 2015). While it could be argued that EYFP or GCaMP3 labeling in *Gipr*-Cre mice could result in part from lineage tracing, we observed a similar location of *Gipr*-positive cells using RNAscope. The activation of *hSyn*-DIO-hM3D(G_q)-mCherry in the DREADD experiments provides further proof that Cre, and by implication, *Gipr*, is actively transcribed in adult hypothalamic cells.

To identify whether *Gipr* cells could be assigned to known neural networks, we performed scRNA-seq, which revealed substantial heterogeneity of *Gipr* cells. Although some genes are likely to have exhibited altered expression during the time taken for cell dissociation and separation, the results allowed us to cluster *Gipr* cells into several populations, including neurons, mural cells, ependymocytes, VLMCs, and oligodendrocytes, each characterized by a distinct profile of marker genes. *Gipr* neurons near-ubiquitously expressed *Sst*, with many also expressing *Avp* and *Pthlh* and fewer expressing *Cartpt*, *Tac1* and *Pomc*. The broad expression of *Sst* in *Gipr* neurons is striking, and the co-expression of *Pthlh* in 50% of *Gipr* neurons suggests that they may predominantly belong to the *Pthlh* clade of SST neurons identified in recent studies (Campbell et al., 2017).

Collective stimulation of *Gipr* cells in the hypothalamus via chemogenetic activation resulted in acute anorexia. While the decrease in food intake upon acute activation of hypothalamic *Gipr* cells appears to be at odds with the protection of *Gipr* KO animals from diet-induced obesity, it is likely that the resistance to weight gain exhibited by *Gipr* KO mice is at least in part due to

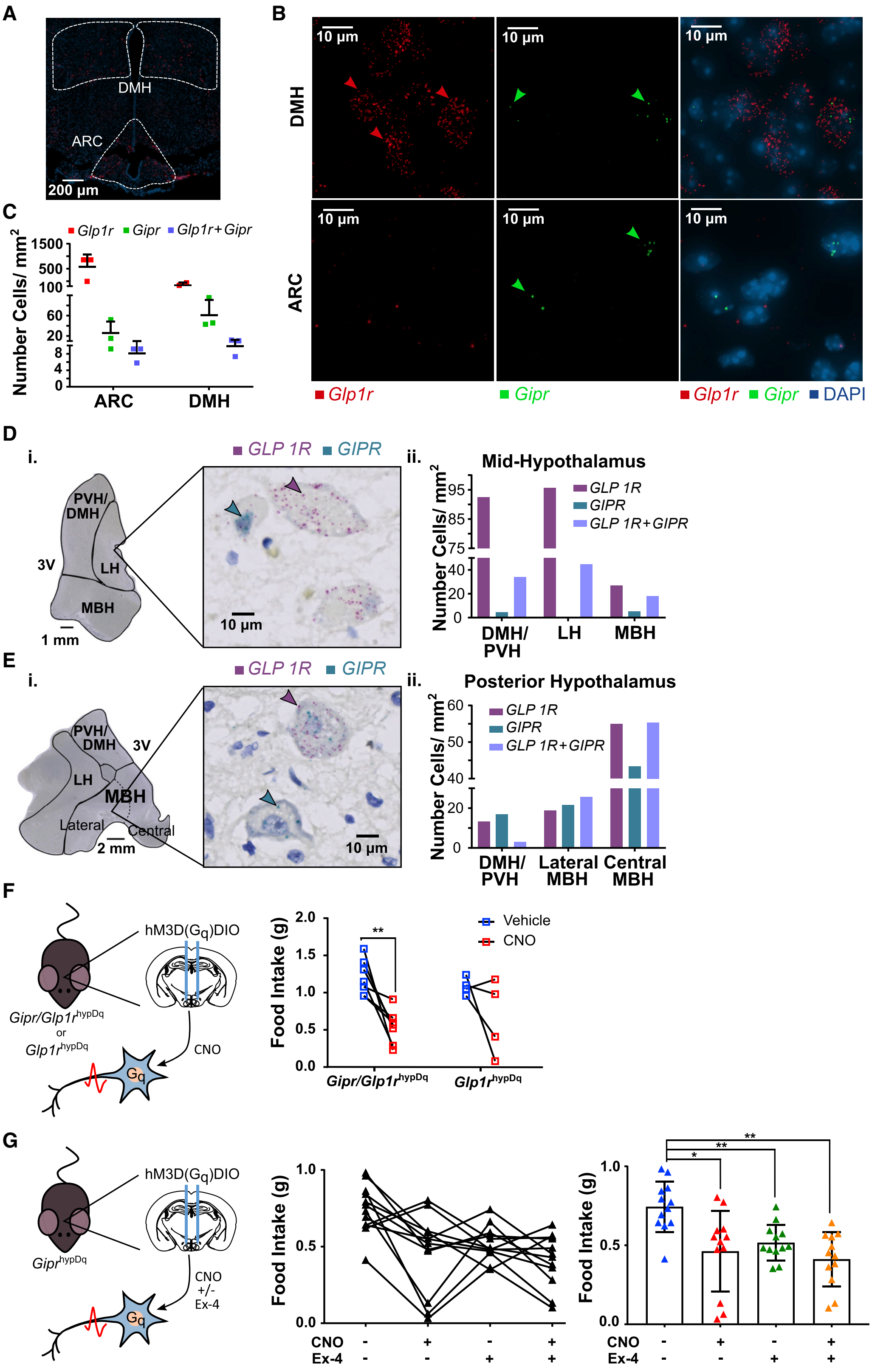

(legend on next page)

decreased insulin signaling (Campbell et al., 2016). Indeed, *Glp1r* KO mice are similarly protected against HFD (Ayala et al., 2010; Hansotia et al., 2007), and although GLP1R agonists undoubtedly suppress food intake, GLP1R inhibition has been linked to increased energy expenditure under HFD conditions (Krieger et al., 2018), indicating that incretin hormones have a complex role in regulating appetite and adiposity.

Suppression of food intake in *Gipr*^{hypDq} mice was robust in the dark phase, when appetite-promoting signaling is at its highest in rodents (Yannielli et al., 2007). Suppression of food intake mediated by hypothalamic *Gipr* cells would be compatible with expression of established anorexic neuropeptides, in particular AVP and CART (Pei et al., 2014), (Farzi et al., 2018), which were expressed in over 40% of *Gipr* neurons. While it may be tempting to speculate that the melanocortin axis may play a role in *Gipr* cell-mediated anorexia, *Pomc* was only expressed in a minority of *Gipr* neurons and at relatively low levels (Figure 2E). Still, it is surprising that the majority of *Gipr* neurons expressed *Sst*, as activation of all five *Sst*-positive neuronal clades (defined by co-expression of *Th*, *Nts*, *Agrp*, *Unc13c*, or *Pthlh*) with D_q in the ARC has been shown to be orexigenic (Campbell et al., 2017). By contrast, our results suggest that specific activation of *Sst*-positive subpopulations expressing *Gipr* (being *Pthlh* positive, but *Agrp* negative) results in anorexia. Although we are not able to link the clear anorexigenic effects seen with D_q activation to a defined cell population, the use of *hSyn*-promoter AAV8 would suggest neuronal targeting (Hammond et al., 2017), though further experiments are required to determine exactly which *Gipr* cell types underlie the observed inhibition of food intake.

The synergy between pharmacological GIPR and GLP1R agonism on appetite (Coskun et al., 2018; Finan et al., 2013; Nørregaard et al., 2018) suggests that the GIPR signaling cascade may act to sensitize hypothalamic cells to other anorectic signals. GLP-1 suppresses appetite partly via centrally expressed GLP1R (Secher et al., 2014). As our RNAscope analysis revealed expression of *Gipr* and *Glp1r* in distinct as well as overlapping cell populations in the hypothalamus, the beneficial metabolic effects of GIP and GLP-1 receptor co-activation could arise from synergistic activity of the two receptors on the same cells or from integration of signals downstream of activating different neuronal populations. The lack of a clear additive effect of GLP1R co-activation on acute anorexia downstream of *Gipr* cell D_q activation may suggest that chronic GIPR activation is needed to potentiate the anorexigenic effects of GLP1R agonism. Our identification of *Gipr* in non-neuronal cells, including cells potentially contributing to the blood brain barrier, raises the possibility that GIPR activation might also alter the accessibility of GLP-1 and other circulating hormones to central nuclei. Future work should address the importance of such mechanisms for chronic GIPR agonist treatments.

In summary, we have characterized previously unrecognized populations of hypothalamic cells that express *Gipr* in rodents and humans and demonstrated that their acute stimulation potently reduces food intake, identifying the central hypothalamic GIP signaling axis as an additional contributor to the control of energy homeostasis.

Limitations of Study

We report a new *Gipr*-Cre knockin mouse model to characterize and manipulate hypothalamic cells. While a knockin model is less likely to result in aberrant Cre expression than transgenic models employing randomly integrated constructs in which a gene promoter (often of limited length) drives a transgene, we cannot exclude that some cells might report Cre activity even in the absence of physiologically relevant *Gipr* expression. This could also result through lineage tracing, where cells are reported that only transiently expressed *Gipr*, although the finding that DIO-AAVs were activated in the adult hypothalamus indicates ongoing Cre expression, mirroring the detection of *Gipr* mRNA by RNAscope. We did not observe Ca^{2+} responses to GIP in primary cultured neurons, however, GIPR is predominantly G_s coupled, and therefore, we would not expect that it's activation would result in increased intracellular calcium levels. Similarly, we do not see acute Ca^{2+} elevation in *Glp1r*-positive neurons in response to GLP-1, which also predominantly activates G_s rather than G_q signaling. It should also be noted that activation of neurons with D_q does not replicate native G_s-coupled activation of GIPR. However, transgenic overexpression of GIP elicited a marked reduction in energy intake (Kim et al., 2012), and ICV administration of GIP reduced food intake (NamKoong et al., 2017). These data, combined with a recent report using a potent GIPR agonist (Mroz et al., 2019), demonstrate the potential for native GIPR signaling to impact feeding behavior. Further work should address the differences of G_s versus G_q activation in *Gipr* neurons and the role of non-neuronal cells in GIPR signaling in the brain. As we were unable to detect an additive effect of GLP1R and hypothalamic *Gipr* cell activation on food intake, future work should also address the role of *Gipr* cells beyond the hypothalamus as well as the metabolic outcomes of chronic rather than acute *Gipr* cell activation.

Figure 4. Partial Cellular Overlap of *Gipr* and *Glp1r* Expression, but Limited Effect of GLP1R-Co-activation on *Gipr*-Expressing Cell-Mediated Acute Anorexia

(A–E) Coronal sections of mouse (A–C) and human (D–E) hypothalamus were co-labeled for *Gipr* or *GIPR* and *Glp1r* or *GLP1R* mRNA using RNAscope. Areas corresponding to the ARC and DMH in mouse and PVH/DMH, lateral hypothalamus (LH), and mediobasal hypothalamus (MBH) in human were assessed for *Gipr* or *GIPR* and *Glp1r* or *GLP1R* expression (B), (Di), and (Ei). Single- and double-labeled cells were counted and scored (C), (Dii), and (Eii.). Bars represent the mean ± SD (see also Figure S4).

(F) *Gipr*-Cre x *Glp1r*-Cre and *Glp1r*-Cre-only mice were injected bilaterally with AAV-DIO-hM3D-mCherry into the hypothalamus to produce *Gipr/Glp1r*^{hypDq} and *Glp1r*^{hypDq} mice, respectively. CNO (1 mg/kg) or vehicle was injected i.p. following a 10-h daytime fast at the onset of the dark phase before measuring food intake 2 h post-activation (see also Figures S3C and S3D). Food intake was compared using a repeated measures 2-way ANOVA with a Sidak's post-hoc test. $^{**}p < 0.01$, *Gipr/Glp1r*^{hypDq} n = 7, *Glp1r*^{hypDq} n = 4.

(G) Heterozygous *Gipr*-Cre mice were injected bilaterally with AAV-DIO-hM3D-mCherry into the hypothalamus to produce *Gipr*^{hypDq} mice. Following a 10-h daytime fast Exendin-4 (Ex-4) (1.5 nmol/kg) or saline was injected s.c. 1 h prior to the onset of the dark phase. CNO (0.3 mg/kg) or vehicle was injected i.p. at the onset of the dark phase, food was presented, and food intake measurements were taken 2 h post-activation (see also Figures S3E, S4C, and S4D). Bars represent mean ± SD. Food intake was compared using a repeated measures 2-way ANOVA with a Sidak's post-hoc test. $^{*}p < 0.05$, $^{**}p < 0.01$, *Gipr*^{hypDq} n = 12.

STAR★METHODS

Detailed methods are provided in the online version of this paper and include the following:

- KEY RESOURCES TABLE
- LEAD CONTACT AND MATERIALS AVAILABILITY
- EXPERIMENTAL MODEL AND SUBJECT DETAILS
  - Human Subjects
  - Animals
  - Primary Culture of Hypothalamic Neurons
- METHOD DETAILS
  - Body Composition Analysis
  - Immunohistochemistry
  - Flow Cytometry
  - Single Cell RNA Sequencing
  - Quantitative RT-PCR
  - Calcium Imaging
  - Viral Injections
  - Food Intake Measurements
  - RNAscope
- QUANTIFICATION AND STATISTICAL ANALYSIS
  - Data Analysis
- DATA AND CODE AVAILABILITY

SUPPLEMENTAL INFORMATION

Supplemental Information can be found online at https://doi.org/10.1016/j.cmet.2019.07.013.

ACKNOWLEDGMENTS

Metabolic Research Laboratories support was provided by the following core facilities: Disease Model Core, Genomics and Transcriptomics Core, Histology Core, Imaging Core, and Core Biochemical Assay Laboratory (supported by the MRC [MRC_MC_UU_12012/5] and Wellcome Trust [100574/Z/12/Z]). Embryos injections to generate *Gipr*-Cre mice were performed by Debbie Drage at Central Biomedical Services. RNA-sequencing was undertaken at the CRUK Cambridge Institute Genomics Core. Cell sorting was performed at the NIHR Cambridge BRC Cell Phenotyping Hub. We thank the histopathology/ISH core facility at Cancer Research UK- Cambridge Institute, in particular Julia Jones for assistance with *in situ* hybridization. The authors thank Eli Lilly and Company, especially Ricardo Samms, for helpful discussion. Research in the laboratory of F.M.G. and F.R. is supported by the MRC (MRC_MC_UU_12012/3) and Wellcome Trust (106262/Z/14/Z and 106263/Z/14/Z). E.K.B. was supported by a MedImmune PhD studentship. F.M.G. and F.R. act as guarantors for this manuscript.

AUTHOR CONTRIBUTIONS

A.E.A., E.K.B., J.T., F.R., F.M.G., and C.B. designed research studies. A.E.A., E.K.B., T.D., T.S., M.G., J.P.-W., W.P., and C.B. conducted experiments. A.E.A., D.C., and B.Y.L. performed data/bioinformatics analysis. I.Z. and F.R. developed transgenic models. F.R., F.M.G., and G.S.H.Y. supervised the project. A.E.A., F.R., and F.M.G. wrote the manuscript.

DECLARATION OF INTERESTS

F.M.G. is a paid consultant for Kallyope, New York. The Gribble-Reimann lab hosts projects that receive funding from MedImmune/AstraZeneca (F.M.G./F.R.). The F.R./F.M.G. laboratory has recently agreed a collaboration with Lilly on future work in the mechanism of GIPR activation. J.P.-W. joined Novo Nordisk (DK) and E.K.B. joined Absolute Antibody (UK) after completing their contributions to this manuscript while working at IMS. There are no other conflicts of interest to declare.

Received: December 21, 2018
Revised: June 28, 2019
Accepted: July 29, 2019
Published: August 22, 2019

REFERENCES

Armbruster, B.N., Li, X., Pausch, M.H., Herlitze, S., and Roth, B.L. (2007). Evolving the lock to fit the key to create a family of G protein-coupled receptors potently activated by an inert ligand. Proc. Natl. Acad. Sci. USA *104*, 5163–5168.

Ayala, J.E., Bracy, D.P., James, F.D., Burmeister, M.A., Wasserman, D.H., and Drucker, D.J. (2010). Glucagon-like peptide-1 receptor knockout mice are protected from high-fat diet-induced insulin resistance. Endocrinology *151*, 4678–4687.

Boylan, M.O., Glazebrook, P.A., Tatalovic, M., and Wolfe, M.M. (2015). Gastric inhibitory polypeptide immunoneutralization attenuates development of obesity in mice. Am. J. Physiol. Endocrinol. Metab. *309*, E1008–E1018.

Buchan, A.M., Polak, J.M., Capella, C., Solcia, E., and Pearse, A.G. (1978). Electronimmunocytochemical evidence for the K cell localization of gastric inhibitory polypeptide (GIP) in man. Histochemistry *56*, 37–44.

Buffa, R., Polak, J.M., Pearse, A.G., Solcia, E., Grimelius, L., and Capella, C. (1975). Identification of the intestinal cell storing gastric inhibitory peptide. Histochemistry *43*, 249–255.

Butler, A., Hoffman, P., Smibert, P., Papalexi, E., and Satija, R. (2018). Integrating single-cell transcriptomic data across different conditions, technologies, and species. Nat. Biotechnol. *36*, 411–420.

Campbell, J.E., and Drucker, D.J. (2013). Pharmacology, physiology, and mechanisms of incretin hormone action. Cell Metab. *17*, 819–837.

Campbell, J.E., Ussher, J.R., Mulvihill, E.E., Kolic, J., Baggio, L.L., Cao, X., Liu, Y., Lamont, B.J., Morii, T., Streutker, C.J., et al. (2016). TCF1 links GIPR signaling to the control of beta cell function and survival. Nat. Med. *22*, 84–90.

Campbell, J.N., Macosko, E.Z., Fenselau, H., Pers, T.H., Lyubetskaya, A., Tenen, D., Goldman, M., Verstegen, A.M., Resch, J.M., McCarroll, S.A., et al. (2017). A molecular census of arcuate hypothalamus and median eminence cell types. Nat. Neurosci. *20*, 484–496.

Chen, R., Wu, X., Jiang, L., and Zhang, Y. (2017). Single-cell RNA-Seq reveals hypothalamic cell diversity. Cell Rep. *18*, 3227–3241.

Coskun, T., Sloop, K.W., Loghin, C., Alsina-Fernandez, J., Urva, S., Bokvist, K.B., Cui, X., Briere, D.A., Cabrera, O., Roell, W.C., et al. (2018). LY3298176, a novel dual GIP and GLP-1 receptor agonist for the treatment of type 2 diabetes mellitus: from discovery to clinical proof of concept. Mol. Metab. *18*, 3–14.

DiMarchi, R.D. (2018). "Let's stay together"; GIP and GLP-1 dual agonism in the treatment of metabolic disease. Mol. Metab. *18*, 1–2.

Dupre, J., Ross, S.A., Watson, D., and Brown, J.C. (1973). Stimulation of insulin secretion by gastric inhibitory polypeptide in man. J. Clin. Endocrinol. Metab. *37*, 826–828.

Eckel, R.H., Fujimoto, W.Y., and Brunzell, J.D. (1979). Gastric inhibitory polypeptide enhanced lipoprotein lipase activity in cultured preadipocytes. Diabetes *28*, 1141–1142.

Elliott, R.M., Morgan, L.M., Tredger, J.A., Deacon, S., Wright, J., and Marks, V. (1993). Glucagon-like peptide-1 (7–36)amide and glucose-dependent insulinotropic polypeptide secretion in response to nutrient ingestion in man: acute post-prandial and 24-h secretion patterns. J. Endocrinol. *138*, 159–166.

Faivre, E., Gault, V.A., Thorens, B., and Hölscher, C. (2011). Glucose-dependent insulinotropic polypeptide receptor knockout mice are impaired in learning, synaptic plasticity, and neurogenesis. J. Neurophysiol. *105*, 1574–1580.

Farzi, A., Lau, J., Ip, C.K., Qi, Y., Shi, Y.C., Zhang, L., Tasan, R., Sperk, G., and Herzog, H. (2018). Arcuate nucleus and lateral hypothalamic CART neurons in the mouse brain exert opposing effects on energy expenditure. Elife *7*.

Finan, B., Ma, T., Ottaway, N., Müller, T.D., Habegger, K.M., Heppner, K.M., Kirchner, H., Holland, J., Hembree, J., Raver, C., et al. (2013). Unimolecular dual incretins maximize metabolic benefits in rodents, monkeys, and humans. Sci. Transl. Med. *5*, 209ra151.

Finan, B., Müller, T.D., Clemmensen, C., Perez-Tilve, D., DiMarchi, R.D., and Tschöp, M.H. (2016). Reappraisal of GIP pharmacology for metabolic diseases. Trends Mol. Med. *22*, 359–376.

Frias, J.P., Nauck, M.A., Van, J., Kutner, M.E., Cui, X., Benson, C., Urva, S., Gimeno, R.E., Milicevic, Z., Robins, D., et al. (2018). Efficacy and safety of LY3298176, a novel dual GIP and GLP-1 receptor agonist, in patients with type 2 diabetes: a randomised, placebo-controlled and active comparator-controlled phase 2 trial. Lancet *392*, 2180–2193.

Fulurija, A., Lutz, T.A., Sladko, K., Osto, M., Wielinga, P.Y., Bachmann, M.F., and Saudan, P. (2008). Vaccination against GIP for the treatment of obesity. PLoS One *3*, e3163.

Hammond, S.L., Leek, A.N., Richman, E.H., and Tjalkens, R.B. (2017). Cellular selectivity of AAV serotypes for gene delivery in neurons and astrocytes by neonatal intracerebroventricular injection. PLoS One *12*, e0188830.

Hansotia, T., Maida, A., Flock, G., Yamada, Y., Tsukiyama, K., Seino, Y., and Drucker, D.J. (2007). Extrapancreatic incretin receptors modulate glucose homeostasis, body weight, and energy expenditure. J. Clin. Invest. *117*, 143–152.

He, L., Vanlandewijck, M., Raschperger, E., Andaloussi Mäe, M., Jung, B., Lebouvier, T., Ando, K., Hofmann, J., Keller, A., and Betsholtz, C. (2016). Analysis of the brain mural cell transcriptome. Sci. Rep. *6*, 35108.

Heeley, N., Kirwan, P., Darwish, T., Arnaud, M., Evans, M.L., Merkle, F.T., Reimann, F., Gribble, F.M., and Blouet, C. (2018). Rapid sensing of l-leucine by human and murine hypothalamic neurons: neurochemical and mechanistic insights. Mol. Metab. *10*, 14–27.

Kaplan, A.M., and Vigna, S.R. (1994). Gastric inhibitory polypeptide (GIP) binding sites in rat brain. Peptides *15*, 297–302.

Killion, E., Wang, J., Yie, J., Shi, S., Bates, D., Min, X., Komorowski, R., Hager, T., Deng, L., Atangan, L., et al. (2018). Anti-obesity effects of GIPR agonists alone and in combination with GLP-1R agonists in preclinical models. Science Transl. Med. *10*.

Kim, S.J., Nian, C., Karunakaran, S., Clee, S.M., Isales, C.M., and McIntosh, C.H. (2012). GIP-overexpressing mice demonstrate reduced diet-induced obesity and steatosis, and improved glucose homeostasis. PLoS One *7*, e40156.

Krieger, J.P., Langhans, W., and Lee, S.J. (2018). Novel role of GLP-1 receptor signaling in energy expenditure during chronic high fat diet feeding in rats. Physiol. Behav. *192*, 194–199.

Kügler, S., Kilic, E., and Bähr, M. (2003). Human synapsin 1 gene promoter confers highly neuron-specific long-term transgene expression from an adenoviral vector in the adult rat brain depending on the transduced area. Gene Ther. *10*, 337–347.

Lam, B.Y.H., Cimino, I., Polex-Wolf, J., Nicole Kohnke, S., Rimmington, D., Iyemere, V., Heeley, N., Cossetti, C., Schulte, R., Saraiva, L.R., et al. (2017). Heterogeneity of hypothalamic pro-opiomelanocortin-expressing neurons revealed by single-cell RNA sequencing. Mol. Metab. *6*, 383–392.

Luche, H., Weber, O., Nageswara Rao, T., Blum, C., and Fehling, H.J. (2007). Faithful activation of an extra-bright red fluorescent protein in "knock-in" Cre-reporter mice ideally suited for lineage tracing studies. Eur. J. Immunol. *37*, 43–53.

Marques, S., Zeisel, A., Codeluppi, S., van Bruggen, D., Mendanha Falcão, A., Xiao, L., Li, H., Häring, M., Hochgerner, H., Romanov, R.A., et al. (2016). Oligodendrocyte heterogeneity in the mouse juvenile and adult central nervous system. Science *352*, 1326–1329.

McClean, P.L., Irwin, N., Cassidy, R.S., Holst, J.J., Gault, V.A., and Flatt, P.R. (2007). GIP receptor antagonism reverses obesity, insulin resistance, and associated metabolic disturbances induced in mice by prolonged consumption of high-fat diet. Am. J. Physiol. Endocrinol. Metab. *293*, E1746–E1755.

Miyawaki, K., Yamada, Y., Ban, N., Ihara, Y., Tsukiyama, K., Zhou, H., Fujimoto, S., Oku, A., Tsuda, K., Toyokuni, S., et al. (2002). Inhibition of gastric inhibitory polypeptide signaling prevents obesity. Nat. Med. *8*, 738–742.

Mroz, P.A., Finan, B., Gelfanov, V., Yang, B., Tschöp, M.H., DiMarchi, R.D., and Perez-Tilve, D. (2019). Optimized GIP analogs promote body weight lowering in mice through GIPR agonism no antagonism. Mol. Metab. *20*, 51–62.

NamKoong, C., Kim, M.S., Jang, B.T., Lee, Y.H., Cho, Y.M., and Choi, H.J. (2017). Central administration of GLP-1 and GIP decreases feeding in mice. Biochem. Biophys. Res. Commun. *490*, 247–252.

Nauck, M.A., Heimesaat, M.M., Orskov, C., Holst, J.J., Ebert, R., and Creutzfeldt, W. (1993). Preserved incretin activity of glucagon-like peptide 1 [7–36 amide] but not of synthetic human gastric inhibitory polypeptide in patients with type-2 diabetes mellitus. J. Clin. Invest. *91*, 301–307.

Nørregaard, P.K., Deryabina, M.A., Tofteng Shelton, P., Fog, J.U., Daugaard, J.R., Eriksson, P.O., Larsen, L.F., and Jessen, L. (2018). A novel GIP analogue, ZP4165, enhances glucagon-like peptide-1-induced body weight loss and improves glycaemic control in rodents. Diabetes Obes. Metab. *20*, 60–68.

Nyberg, J., Anderson, M.F., Meister, B., Alborn, A.M., Ström, A.K., Brederlau, A., Illerskog, A.C., Nilsson, O., Kieffer, T.J., Hietala, M.A., et al. (2005). Glucose-dependent insulinotropic polypeptide is expressed in adult hippocampus and induces progenitor cell proliferation. J. Neurosci. *25*, 1816–1825.

Paratore, S., Ciotti, M.T., Basille, M., Vaudry, D., Gentile, A., Parenti, R., Calissano, P., and Cavallaro, S. (2011). Gastric inhibitory polypeptide and its receptor are expressed in the central nervous system and support neuronal survival. Cent. Nerv. Syst. Agents Med. Chem. *11*, 210–222.

Pei, H., Sutton, A.K., Burnett, K.H., Fuller, P.M., and Olson, D.P. (2014). AVP neurons in the paraventricular nucleus of the hypothalamus regulate feeding. Mol. Metab. *3*, 209–215.

Ravn, P., Madhurantakam, C., Kunze, S., Matthews, E., Priest, C., O'Brien, S., Collinson, A., Papworth, M., Fritsch-Fredin, M., Jermutus, L., et al. (2013). Structural and pharmacological characterization of novel potent and selective monoclonal antibody antagonists of glucose-dependent insulinotropic polypeptide receptor. J. Biol. Chem. *288*, 19760–19772.

Richards, P., Parker, H.E., Adriaenssens, A.E., Hodgson, J.M., Cork, S.C., Trapp, S., Gribble, F.M., and Reimann, F. (2014). Identification and characterization of GLP-1 receptor-expressing cells using a new transgenic mouse model. Diabetes *63*, 1224–1233.

Secher, A., Jelsing, J., Baquero, A.F., Hecksher-Sørensen, J., Cowley, M.A., Dalbøge, L.S., Hansen, G., Grove, K.L., Pyke, C., Raun, K., et al. (2014). The arcuate nucleus mediates GLP-1 receptor agonist liraglutide-dependent weight loss. J. Clin. Invest. *124*, 4473–4488.

Smith, M.A., Katsouri, L., Virtue, S., Choudhury, A.I., Vidal-Puig, A., Ashford, M.L.J., and Withers, D.J. (2018). Calcium channel CaV2.3 subunits regulate hepatic glucose production by modulating leptin-induced excitation of arcuate pro-opiomelanocortin neurons. Cell Rep. *25*, 278–287.e4.

Sparre-Ulrich, A.H., Hansen, L.S., Svendsen, B., Christensen, M., Knop, F.K., Hartmann, B., Holst, J.J., and Rosenkilde, M.M. (2015). Species-specific action of (Pro3)GIP - an efficacious agonist on human GIP receptor, but partial agonist and competitive antagonist on rat and mouse GIP receptors. Br. J. Pharmacol.

Turton, M.D., O'Shea, D., Gunn, I., Beak, S.A., Edwards, C.M., Meeran, K., Choi, S.J., Taylor, G.M., Heath, M.M., Lambert, P.D., et al. (1996). A role for glucagon-like peptide-1 in the central regulation of feeding. Nature *379*, 69–72.

Ugleholdt, R., Pedersen, J., Bassi, M.R., Füchtbauer, E.M., Jørgensen, S.M., Kissow, H.L., Nytofte, N., Poulsen, S.S., Rosenkilde, M.M., Seino, Y., et al. (2011). Transgenic rescue of adipocyte glucose-dependent insulinotropic polypeptide receptor expression restores high fat diet-induced body weight gain. J. Biol. Chem. *286*, 44632–44645.

Usdin, T.B., Mezey, E., Button, D.C., Brownstein, M.J., and Bonner, T.I. (1993). Gastric inhibitory polypeptide receptor, a member of the secretin-vasoactive intestinal peptide receptor family, is widely distributed in peripheral organs and the brain. Endocrinology *133*, 2861–2870.

Wasada, T., McCorkle, K., Harris, V., Kawai, K., Howard, B., and Unger, R.H. (1981). Effect of gastric inhibitory polypeptide on plasma levels of chylomicron triglycerides in dogs. J. Clin. Invest. *68*, 1106–1107.

Waterson, M.J., and Horvath, T.L. (2015). Neuronal regulation of energy homeostasis: beyond the hypothalamus and feeding. Cell Metab. *22*, 962–970.

Yannielli, P.C., Molyneux, P.C., Harrington, M.E., and Golombek, D.A. (2007). Ghrelin effects on the circadian system of mice. J. Neurosci. *27*, 2890–2895.

Zariwala, H.A., Borghuis, B.G., Hoogland, T.M., Madisen, L., Tian, L., De Zeeuw, C.I., Zeng, H., Looger, L.L., Svoboda, K., and Chen, T.W. (2012). A Cre-dependent GCaMP3 reporter mouse for neuronal imaging in vivo. J. Neurosci. *32*, 3131–3141.

STAR★METHODS

KEY RESOURCES TABLE

REAGENT or RESOURCE	SOURCE	IDENTIFIER
Antibodies		
Goat Polyclonal anti-GLP-1	Santa Cruz Biotechnology	Cat # sc-7782; RRID: AB_2107325
Guinea Pig Polycloal anti-insulin	Abcam	Cat # 7842; RRID: AB_306130
Goat Polyclonal anti-GFP	Abcam	Cat # 5450; RRID: AB_304897
Rabbit Polyclonal anti-DsRed	Takara Bio	Cat # 632496; RRID: AB_10013483
Alexa 488 secondary anti-goat	Thermo Fisher Scientific	Cat # A32814; RRID: AB_2762838
Alexa 555 secondary anti-rabbit	Thermo Fisher Scientific	Cat # A32794; RRID: AB_2762834
Alexa 633 secondary anti-guinea pig	Thermo Fisher Scientific	Cat # A-21105; RRID: AB_2535757
Biotinylated donkey anti-goat IgG	Millipore	Cat # AP180B; RRID: AB_11214009
Bacterial and Virus Strains		
AAV-hSyn-DIO-hM3D(Gq)-mCherry	Addgene	Cat # 44361-AAV8
Biological Samples		
Human hypothalamic brain blocks	Cambridge Brain Bank	https://www.cuh.nhs.uk/for-public/cambridge-brain-bank
Chemicals, Peptides, and Recombinant Proteins		
Papain	Worthingon/ Lorne Labs	Cat # LK003178
SuperScript III Reverse Transcriptase	Thermo Fisher Scientific	Cat # 18080093
SuperScript II Reverse Transcriptase	Thermo Fisher Scientific	Cat # 18064014
TaqMan Fast Universal PCR Master Mix	Thermo Fisher Scientific	Cat # 4364103
Glutamate	Sigma	Cat # G1251
CCK (octapeptide, sulfated)	Tocris	Cat # 1166
Hexarelin	LKT Laboratories	Cat # H1893
Leptin	R&D Systems	Cat # 498-OB-01M
5-HT	Sigma	Cat # H9523
Clozapine-N-Oxide	Sigma	Cat # C0832
Exendin-4	Tocris	Cat # 1933
Critical Commercial Assays		
RNeasy Micro Kit	QIAGEN	Cat # 74004
RNeasy Plus Micro Kit	QIAGEN	Cat # 74034
10× Genomics Chromium Single Cell Library Kit v2	10× Genomics	Cat # 120234
RNAscope® 2.5 LS Multiplex Reagent Kit	Advanced Cell Diagnostics	Cat # 322800
RNAscope® LS 2.5 Probe- Mm-Gipr	Advanced Cell Diagnostics	Cat # 319128
RNAscope® 2.5 LS Probe- Mm-Glp1r	Advanced Cell Diagnostics	Cat # 418858
RNAscope® 3-plex LS Multiplex Control Positive Probe- Mm polr2A, ppib, ubc	Advanced Cell Diagnostics	Cat # 320888
RNAscope® 3-plex LS Multiplex Negative Control Probe- dapB	Advanced Cell Diagnostics	Cat # 320878
RNAscope® 2.5 LS Duplex Reagent Kit	Advanced Cell Diagnostics	Cat # 322440
RNAscope® LS 2.5 Probe- Hs-GLP1R	Advanced Cell Diagnostics	Cat # 519828
RNAscope® 2.5 LS Probe- Hs-GIPR	Advanced Cell Diagnostics	Cat # 471348
RNAscope® 2.5 LS Positive Control Probe- Hs-PPIB	Advanced Cell Diagnostics	Cat # 313908
RNAscope® 2.5 LS Duplex Negative Control Probe- DapB, DapB	Advanced Cell Diagnostics	Cat # 320758

(*Continued on next page*)

Continued		
REAGENT or RESOURCE	SOURCE	IDENTIFIER
Deposited Data		
scRNAseq data from hypothalamic *Gipr*-expressing cells isolated from *Gipr*-Cre mice	NCBI GEO	GSE134726
Experimental Models: Organisms/Strains		
Gipr-Cre Mice	This paper	N/A
Glp1r-Cre Mice	Richards et al. (2014)	N/A
ROSA26-EYFP Cre Reporter Mice	Derived from JAX:B6.129X1-Gt(ROSA) $26Sor^{tm1(EYFP)Cos}$/J	N/A
ROSA26-GCaMP3 Cre Reporter Mice	Gift, presumed derived from JAX:B6; 129S-Gt(ROSA)$26Sor^{tm38(CAG-GCaMP3)Hze}$/J	N/A
Oligonucleotides		
Gipr TaqMan Gene Expression Assay; mm01316344_m1	Thermo Fisher Scientific	Cat # 4448892
Sst TaqMan Gene Expression Assay; mm00436671_m1	Thermo Fisher Scientific	Cat # 4448892
Avp TaqMan Gene Expression Assay; mm00437761_g1	Thermo Fisher Scientific	Cat # 4448892
Pomc TaqMan Gene Expression Assay; mm00435874_m1	Thermo Fisher Scientific	Cat # 4448892
Agrp TaqMan Gene Expression Assay; mm00475829_g1	Thermo Fisher Scientific	Cat # 4448892
Htr2c TaqMan Gene Expression Assay; mm00434127_m1	Thermo Fisher Scientific	Cat # 4448892
Cckbr TaqMan Gene Expression Assay; mm00432329_m1	Thermo Fisher Scientific	Cat # 4448892
Ghsr TaqMan Gene Expression Assay; mm00616415_m1	Thermo Fisher Scientific	Cat # 4448892
Glp1r TaqMan Gene Expression Assay; mm00445292_m1	Thermo Fisher Scientific	Cat # 4448892
iCre FAM/TAMRA primers/probe: probe: 5'(6FAM)TGAAGGACATCTCCC GCACCG(TAM)3', Fwd: 5'CAATGTGGA TCAGCATTCTCC3', Rev: 3'GCCGAAATTGCCAGAATCAG3'	This paper	N/A
Software and Algorithms		
CellRanger Analysis Pipeline v2.0	10X Genomics	https://support.10xgenomics.com/single-cell-gene-expression/software/pipelines/latest/installation
Seurat v2.3.4 R Package	Butler et al. (2018)	http://seurat.r-forge.r-project.org/; RRID: SCR_007322
GraphPad Prism 7.0	GraphPad Software	RRID: SCR_002798
MetaFluor	Molecular Devices/ Cairn Research	http://www.moleculardevices.com/systems/metamorph-research-imaging/metafluor-fluorescence-ratio-imaging-software; RRID: SCR_014294
ZEN Blue	Zeiss	http://www.zeiss.com/microscopy/en_us/products/microscope-software/zen.html#introduction; RRID: SCR_013672
HALO v2.3	Indica Labs	http://www.indicalab.com/halo
HALO FISH v2.1.6 Analysis Module	Indica Labs	http://www.indicalab.com/halo
HALO ISH v2.2 Analysis Module	Indica Labs	http://www.indicalab.com/halo

LEAD CONTACT AND MATERIALS AVAILABILITY

Further information and requests for resources and reagents should be directed to and will be fulfilled by the Lead Contact, Frank Reimann (fr222@cam.ac.uk). *Gipr*-Cre and *Glp1r*-Cre mice are available for collaborations upon reasonable request and will require an MTA before distribution.

EXPERIMENTAL MODEL AND SUBJECT DETAILS

Human Subjects

Anonymised human hypothalamic tissue samples were provided by the Cambridge Brain Bank. Subjects were approached in life for written consent for brain banking, and all tissue donations were collected and stored following legal and ethical guidelines (NHS reference number 11/0EE/0011). Hypothalamic tissue samples from two individuals were used in RNAscope analysis. Both individuals were female—one aged 95 and one aged 86 at the time of death.

Animals

All animal procedures were approved by the University of Cambridge Animal Welfare and Ethical Review Body and conformed to the Animals (Scientific Procedures) Act 1986 Amendment Regulations (SI 2012/3039). The work was performed under the UK Home Office Project Licenses 70/7824, PE50F6065, and PC02F3663. All mice were group-housed and maintained under SPF health/immune status in individually ventilated cages with standard bedding and enrichment unless otherwise stated. Mice were housed in a temperature (24°C) and humidity-controlled room on a 12 h light/dark cycle (lights on 6:00, lights out 18:00) with ad libitum access to water and standard laboratory chow diet (13.3% calories from fat, 22.4 % calories from protein, 64.3% calories from carbohydrate, 3.5 kcal/g; Scientific Animal Food Engineering) unless otherwise stated.

Generation of Mouse Models

Gipr-Cre knock-in mice were generated using CRISPR/Cas9 technology. In brief, initially the *Gipr*-coding sequence from the start codon in exon2 to the stop codon in exon 14 in the bacterial artificial chromosome RP23-384-I23 (Children's Hospital Oakland Research Institute) was replaced with *iCre*-sequence (a generous gift from Rolf Sprengel, Max Planck Institute for Medical Research, Heidelberg, Germany) using Red/ET recombination technology (Genebridges). From this, a 2754bp sequence containing the *iCre* sequence flanked by 816bp and 879bp from the *Gipr* locus was amplified and cloned into pCR-BluntII-TOPO vector to be used as a donor for homologous recombination in C57Bl6/CBA-F1 embryos. One-cell stage fertilized mouse embryos were injected with 15ng/ul circular donor plasmid, 40ng/ul Cas9-protein (ToolGen), 0.61 pmols/ul guide RNAs targeting the wild-type *Gipr* gene (Dharmacon) and 50uM SCR7 inhibitor (Sigma). Positive recombinants were identified by PCR analysis specific for the recombined allele and correct recombination was confirmed by Sanger sequencing. Likely off-target genetic alterations were also sequenced and excluded, even though mice were subsequently crossed with C57Bl6/JN for >8 generations, which will further remove unwanted genetic modifications. Homozygous *Gipr*-Cre mice (= *Gipr* knock-out) were created by crossing heterozygous mice after >8 generations of back-crossing into C57Bl6.

Gipr-Cre mice were crossed with *ROSA26*-EYFP or *ROSA26*-GCaMP3 reporter strains to enable fluorescent detection and intracellular calcium level monitoring of cells expressing *Gipr* by cytosolic EYFP or GCaMP3 expression, respectively (Luche et al., 2007; Zariwala et al., 2012). Reporter strains were on a mixed C57B6J/N genetic background.

To produce mice expressing Cre in both *Gipr and Glp1r* expressing cells, *Gipr*-Cre and *Glp1r*-Cre (Richards et al., 2014) mice were crossed.

Primary Culture of Hypothalamic Neurons

Primary cultures of hypothalamic neurons were prepared from male and female 4 to 6-week-old *Gipr*^{GCaMP3} mice as previously described (Heeley et al., 2018). For each preparation, tissue isolated from two mice was pooled. Mice were killed by cervical dislocation. Brains were extracted, placed into ice-cold Hibernate-A medium (Thermo Fisher Scientific) containing 0.25% GlutaMAX and 2% B27 (Sigma). The hypothalamus was microdissected and placed in extraction media on ice and cut into 1-mm chunks using a scalpel. Tissue was transferred to Hibernate-A minus calcium medium (BrainBits) containing papain (20 U/ml, Worthington) and 1% GlutaMAX (Sigma) pre-heated at 37°C and digested for 30 min at 37°C under agitation (Thermomixer, 500 rpm). After digestion, tissue extracts from 2 animals were pooled, transferred to a tube containing Hibernate-A with 3.5 U/ml DNase I (Sigma) and triturated using a fire-polished glass pipette. The trituration supernatant was gently loaded on top of a BSA gradient prepared in Hibernate-A medium, spun for 5 min at 300 rcf, and the pellet was resuspended in Neurobasal-A medium containing 0.3 nM FGF-Basic (PeproTech, Rocky Hill, NJ, United States of America), 0.25% GlutaMAX (Sigma), and 2% B27 (Sigma). 100 μl of resuspended cells were plated into cloning cylinders (Sigma) on glass bottom 35 mm dishes (MatTek Corporation), coated with poly-lysine (0.1 mg/ml, Sigma). Plates were placed in an incubator (37°C, 5% CO_2) for 1 h. After 1 h, an additional 2 ml culture media was added and the cloning cylinders were removed.

METHOD DETAILS

Body Composition Analysis

Gipr-Cre mice heterozygous for *iCre* at the *Gipr* locus were crossed. At 6-7 weeks of age, the resulting male *Gipr* homozygous, heterozygous, and null offspring were placed on a 45% high fat diet (HFD; 45% calories from fat, 20 % calories from protein,

35% calories from carbohydrate, 4.7 kcal/g; Research Diets Inc.) for 17 weeks. Body weights were recorded twice per week. At the end of 17 weeks on HFD, mice were scanned using a time domain nuclear magnetic resonance (TD-NMR, Bruker Minispec, Bruker Optics, Inc.). The instrument was calibrated for these studies using a quality control check of internal voltages, temperature, magnets, and NMR parameters using a standard provided by the manufacturer.

Immunohistochemistry

Pancreatic and adipose tissues were fixed in 4% paraformaldehyde (PFA), dehydrated in 15% and 30% sucrose and frozen in OCT embedding media (VWR). Cryostat-cut sections (6-10 μm) were mounted directly onto poly-lysine covered glass slides (Thermo Fisher Scientific). Slides were incubated for 1 h in blocking solution containing 5% goat or donkey serum, 0.05% (v/v) Tween-20 and 1% (w/v) BSA. Slides were stained overnight at 4°C with primary antisera in the same blocking solution for proglucagon (1:100, Santa Cruz), insulin (1:100, Abcam), and/or GFP (1:1000, Abcam). Slides were washed with PBS, and incubated with appropriate secondary antisera (donkey or goat AlexaFluors 488 or 555 or 633, Thermo Fisher Scientific) diluted 1:300 for 1 h. Control sections were stained with secondary antisera alone. Sections were mounted with Hydromount (National Diagnostics) prior to confocal microscopy (TCS SP8, Leica).

Brain tissue was collected from perfusion fixed mice. Animals were anaesthetized with Euthatal solution (150 mg/kg in saline) and transcardiacally perfused with PBS followed by 4% PFA. Brains were extracted and post-fixed in 4% PFA, 30% sucrose for 48 h at 4°C. Brains were sectioned using a freezing sliding microtome into 5 subsets of 25 μm sections. For DAB-staining, slices were washed in PBS, then incubated with 0.5% (v/v) hydrogen peroxide for 15 minutes. Slices were washed, then blocked for 1 h in 5% donkey serum, 0.03% (v/v) Tween-20, then incubated with GFP antiserum (1:1000, Abcam) in blocking solution overnight at 4°C. Slices were washed, then incubated in biotinylated donkey anti-goat IgG (1:400, Millipore) in 0.3% (v/v) PBS-Tween20. Sections were incubated with avidin-biotin complex (Vector Laboratories Inc.) and developed using DAB (Abcam). For immunofluorescent staining, slices were washed in PBS, then blocked for 1 h in 5% donkey serum, 0.03% (v/v) Tween-20, then incubated with GFP (1:1000, Abcam) and DsRed antisera (1:1000, Takara Bio) in blocking solution overnight at 4°C. Slices were washed, then incubated with appropriate secondary antisera (AlexaFluors 488 and 555, Thermo Fisher Scientific) diluted 1:300 for 1 h. Sections were then washed, mounted on slides and coverslipped with Vectashield (Vector Laboratories Inc.). Slides were imaged using an Axio Scan.Z1 sliced scanner (Zeiss).

Flow Cytometry

Single cell suspensions were prepared and pooled from the hypothalami of 2-3 *Gipr*^{EYFP} or *Gipr*^{GCaMP3} mice as described previously (Lam et al., 2017). Briefly, mice were sacrificed by cervical dislocation, and tissue from the hypothalamus located ventrally caudal of the optical nerve chiasm (~Bregma -0.3 to -2.92 mm) was dissected into Hibernate-A medium without calcium (BrainBits). The tissue was digested with 20 U/ml Papain (Worthington) for 30 min at 37°C, followed by trituration in Hibernate-A medium (Thermo Fisher Scientific) containing 0.005% (w/v) DNase 1 (Worthington). The cell suspension was filtered through a 40 μm cell strainer into a fresh tube.

Fluorescence-activated cell sorting was performed using an Influx Cell Sorter (BD Biosciences). Cells were gated according to cell size (FSC), cell granularity (SSC), FSC pulse-width for singlets, fluorescence at 488 nm/532 nm for EYFP and 647/670 nm for nuclear stain with DraQ5 (Biostatus).

Single Cell RNA Sequencing

3500 purified EYFP-positive cells from two 4 to 6-week old female *Gipr*^{EYFP} mice were purified as described above and pooled for droplet encapsulation. cDNA libraries from purified EYFP-positive cells were generated using the 10× Genomics Chromium Instrument and single-cell expression V2 reagents (10X Genomics). Pooled libraries were sequenced on an Illumina HiSeq 4000 instrument (26-bp first read, 76bp second read), yielding an average of 99000 reads per cell. Library preparation was performed by the Genomics and Transcriptomic Core at the Institute of Metabolic Science. The sequencing was performed at the Genomics Core, Cancer Research UK Cambridge Institute.

Sequencing reads were aligned to the mouse genome (mm10) using CellRanger analysis pipeline V2.0 (10× Genomics). Downstream analyses were performed using the Seurat v2.3.4 R package (Butler et al., 2018). Cells expressing fewer than 200 unique genes were filtered out from the analysis, leaving 2420 cells.

Gene expression measurements for each cell were normalised using a global-scaling method. 2571 highly variable genes were identified using Seurat with default settings. Dimensionality reduction was performed using principle component analysis (PCA) on these variable genes to identify statistically significant ($p<0.05$) PCs for downstream clustering analysis. Clustering was performed using the Seurat default graph-based clustering approach. The resultant six clusters were plotted using t-distributed stochastic neighbour embedding (t-SNE).

Marker genes were identified for all clusters using the Mann-Whitney U test, implemented by the FindAllMarkers function in the Seurat v2.3.4 R package. The top 20 gene markers were cross referenced against other bulk and scRNAseq databases (Campbell et al., 2017; Chen et al., 2017; He et al., 2016; Marques et al., 2016) to assign cell type identities for each cluster.

Quantitative RT-PCR

For quantitative RT-PCR conducted on purified hypothalamic *Gipr*-expressing cells, EYFP- or GCaMP3-positive cells were FACS-purified as described above, and collected into RLT lysis buffer (QIAGEN) before being frozen on dry ice. EYFP/GCaMP3-negative

cells were also collected. Total RNA was extracted using an RNeasy Plus Micro kit (QIAGEN) according to the manufacturer's protocol. DNAse1 treatment was performed using gDNA spin columns (QIAGEN). RNA was reverse transcribed using the SuperScript III Reverse Transcriptase (Thermo Fisher Scientific).

For quantitative RT-PCR performed on homogenates of whole hypothalamic tissue, total RNA from the hypothalami isolated from 3 *Gipr* wildtype animals was extracted using an RNeasy Mini kit (QIAGEN) according to the manufacturer's protocol. RNA was treated with DNAse1 (Invitrogen), and reverse transcribed using the SuperScript II Reverse Transcriptase (Thermo Fisher Scientific).

qPCR was performed with a QuantStudio 7 Real-Time PCR system (Applied Biosystems). The PCR reaction mix consisted of first-strand cDNA template, TaqMan^{TM} gene expression primer/probe mix (Thermo Fisher Scientific), and PCR master mix (Thermo Fisher Scientific). Expression of the selected targets was compared to that of *Actb* measured on the same sample in parallel on the same plate, giving a CT difference (ΔCT) for *Actb* minus the test gene. Statistics were performed on the ΔCT data and only converted to relative expression levels ($2^{\Delta CT}$) for presentation in the figures.

TaqMan^{TM} primers/probes used are listed in the Key Resources Table.

Calcium Imaging

Imaging experiments were performed using Hamamatsu Orca-ER digital camera (Cairn Research) attached to an Olympus IX71 inverted fluorescent microscope with a 40× oil-immersion objective.

Cultured primary hypothalamic cells were imaged 2-16 h after dissociation. Cells were rinsed with standard bath solution (138 mmol/l NaCl, 4.5 mmol/l KCl, 4.2 mmol/l $NaHCO_3$, 1.2 mmol/l NaH_2PO_4, 2.6 mmol/l $CaCl_2$, 1.2 mmol/l $MgCl_2$, 10 mmol/l HEPES and 10 mmol/l glucose, pH 7.4) and allowed to equilibrate for 15 min. *Gipr* cells were identified by their GCaMP3 fluorescence when excited with 488/8 nm and images were taken every 2 seconds using a 75-W xenon arc lamp. Emission was collected using a 510-nm long-pass filter and all images were collected on MetaFluor software (Molecular Devices. Calcium responses to 100 μmol glutamate (Sigma), 100 nmol/l CCK (Tocris), 100 nmol/l hexarelin (LKT Laboratories), 20 μmol/l 5HT (Sigma), and 10 nM leptin (R&D Systems) were recorded as increases in GCaMP3 emission. Responses to 30 mmol/l KCl were used as a positive control.

Recordings were background subtracted and represented as the 488-nm fluorescence intensity. The average GCaMP3 fluorescence intensity was calculated over 10-second time windows for the entirety of the experiment. Responses to test reagents were expressed as fold-changes determined from the peak fluorescence during a 30 second window following the perfusion of the test reagent divided by the average of the baseline taken 30 seconds before and after test reagent application and wash off, respectively. Responses were considered real if they reached a fold defined as those in which the fluorescence change following stimulus addition was at least 1.2 and above-fold.

Viral Injections

All viral brain injections were performed on 8-10 week old male heterozygous *Gipr*-Cre, *Gipr*-Cre x *Glp1r*-Cre, or *Glp1r*-Cre mice, producing *Gipr*^{hypDq}, *Gipr/Glp1r*^{hypDq}, or *Glp1r*^{hypDq} animals, respectively. Surgical procedures were performed under isofluorane anesthesia, and all animals received Metacam prior to the surgery. Mice were stereotactically implanted with bilateral steel guide cannulae (Plastics One) positioned 1 mm above the ARH (A/P: −1.1 mm, D/V: −4.9 mm, lateral: +0.4 mm from Bregma). Bevelled stainless steel injectors (33 gauge, Plastics One) extending 1 mm from the tip of the guide were used for injections, delivering 500 nl AAV-*hSyn*-DIO-hM3D(Gq)-mCherry at 75 nl/min (Addgene # 44361-AAV8, 4×10^{12} vg/mL). Mice were allowed a 2 week recovery period.

Food Intake Measurements

All food intake studies were performed in a crossover manner (Figure S3) on age- matched groups after 1 week recovery and 1 week daily handling acclimatization post-surgery.

For experiments assessing the effect of *Gipr* D_q activation in *Gipr*^{hypDq} mice, animals were singly housed the day before the experiment. Mice were administered 1 mg/kg clozapine-N-oxide (CNO; Sigma) or an equivalent volume of vehicle containing a matched concentration of DMSO (1%). For light phase activation measurements, mice were injected with either CNO or vehicle at 9:00 following a 2 h fast. Food was weighed 1 h, 2 h, 4 h, 8 h, and 24 h post-injection. For dark phase activation measurements, mice were injected with either CNO or vehicle at 18:00 (start of dark cycle) and food was weighed 2 h later. In dark phase activation measurements on fasted animals, mice were fasted for 10 h prior to the injection. This was a crossover design study, and a full trial was complete after mice had received both CNO and vehicle on each testing regime. At least 3 days elapsed between each injection.

For experiments assessing the effect of *Gipr/Glp1r* cell co-D_q activation, *Gipr/Glp1r*^{hypDq} and *Glp1r*^{hypDq} mice were singly housed following surgery. Mice were fasted for 10 h prior to D_q activation. Mice were administered 1 mg/kg CNO or an equivalent volume of vehicle containing a matched concentration of DMSO (1%) at 18:00 (start of dark cycle) and food was weighed 2 h later. A full trial was complete after mice had received both CNO and vehicle. At least 3 days elapsed between each injection.

For experiments assessing the effect of *Gipr* cell D_q activation in addition to Exendin-4 (Ex-4) treatment *Gipr*^{hypDq} mice were singly housed following surgery. Mice were fasted for 10 h prior to D_q activation. 1.5 nmol/kg Ex-4 (Tocris) or saline control was administered subcutaneously 1 h prior to the onset of the dark phase. Mice were administered 0.3 mg/kg CNO or an equivalent volume of vehicle containing DMSO (1%) at 18:00 (start of dark cycle) and food was weighed 2 h later. A full trial was complete after mice had received both CNO and vehicle on both the Ex-4 and saline control backgrounds. At least 3 days elapsed between each testing paradigm.

RNAscope

Mouse

Brain tissue was collected from three mice for RNAscope analysis. Animals were anaesthetized with Euthatal solution (150 mg/kg in saline) and transcardiacally perfused with PBS followed by 4% PFA. Brains were extracted, sectioned into 0.5 cm thick slices and post-fixed in 4% PFA for 4 h before being transferred to 30% sucrose for 48 h at 4°C and then frozen. Coronal sections were cut at 12 μM and stored at -80°C until required.

Simultaneous detection of mouse *Gipr* and *Glp1r* was performed on fixed, frozen sections using Advanced Cell Diagnostics (ACD) RNAscope® 2.5 LS Multiplex Reagent Kit, RNAscope® LS 2.5 Probe- Mm-Gipr, and RNAscope® 2.5 LS Probe- Mm-Glp1r (ACD). Positive [RNAscope® 3-plex LS Multiplex Control Positive Probe - Mm polr2A, ppib, ubc; ACD] and negative [RNAscope® 3-plex LS Multiplex Negative Control Probe dapB; ACD] controls were performed in parallel. Slides were thawed at room temperature for 10 min before baking at 60°C for 45 min. The sections were then post-fixed in pre-chilled 4% PFA for 15 min at 4°C, washed in 3 changes of PBS for 5 min each before dehydration through 50%, 70 & 100% and 100% Ethanol for 5 min each. The slides were air-dried for 5 min before loading onto a Bond Rx instrument (Leica Biosystems). Slides were prepared using the frozen slide delay prior to pre-treatments using Epitope Retrieval Solution 2 (Leica Biosystems) at 95°C for 5 min, and ACD Enzyme from the Multiplex Reagent kit at 40°C for 10 min. Probe hybridisation and signal amplification was performed according to manufacturer's instructions. The following TSA plus fluorphores were used to detect corresponding RNAscope probes using the BondRx platform according to the ACD protocol: Fluorescein (Akoya Biosciences), and Cy5 (Akoya Biosciences) were Slides were then removed from the Bond Rx and mounted using Prolong Diamond (Thermo Fisher Scientific).

Slides were imaged on a CellDiscoverer 7 microscope (Zeiss). Z-stack images with 1.0 μm spacing were taken for a representative slice from each mouse corresponding to -1.34 to -1.84 mm A/P from Bregma using a 25x water immersion objective. Z-stacks were deconvolved and compressed into 2D images using extended depth of focus (EDF) with maximum projection processing (ZEN Blue, Zeiss). EDF images were read into HALO v2.3 (Indica Labs) as .CZI files for analysis. *Gipr* and *Glp1r* positive cells were detected using the HALO FISH v2.1.6 analysis module based on intensity thresholds set using negative controls for both the fluorescein and Cy5 channels. Cells detected as positive for *Gipr* or *Glp1r* were checked by eye, and were only included in final analysis if there were 2 or more spots corresponding to *Gipr* mRNA, and/or 3 or more spots corresponding to *Glp1r* mRNA.

Human

Simultaneous detection of Human *GLP1R* and *GIPR* was performed on FFPE sections using Advanced Cell Diagnostics (ACD) RNAscope® 2.5 LS Duplex Reagent Kit, RNAscope® LS 2.5 Probe- Hs-GLP1R and RNAscope® 2.5 LS Probe- Hs-GIPR- (ACD, Hayward, CA, USA). Positive (RNAscope® 2.5 LS Positive Control Probe_Hs-PPIB) and negative (RNAscope® 2.5 LS Duplex Negative Control Probe DapB, DapB) controls were performed in parallel (ACD, Hayward, CA, USA). Briefly, sections were baked for 1 h at 60°C before loading onto a Bond RX instrument (Leica Biosystems). Slides were deparaffinized and rehydrated on board before pre-treatments using Epitope Retrieval Solution 2 (Leica Biosystems) at 88°C for 10 minutes, and ACD Enzyme from the Duplex Reagent kit at 40°C for 10 minutes. Probe hybridisation and signal amplification was performed according to manufacturer's instructions. Fast red detection of human *GLP1R* was performed on the Bond Rx using the Bond Polymer Refine Red Detection Kit (Leica Biosystems) according to ACD protocol. Slides were then removed from the Bond Rx and detection of the human *GIPR* signal was performed using the RNAscope® 2.5 LS Green Accessory Pack (ACD) according to kit instructions. Controls were detected using both the fast red and green detection kits. Slides were heated at 60°C for 1 h, dipped in Xylene and mounted using VectaMount Permanent Mounting Medium (Vector Laboratories).

Slides were imaged on a Slide Scanner Axio Scan.Z1 microscope (Zeiss). Images were taken in regions where positive cells were detected using a 40x air objective and sharpened using the Unsharp Masking processing in ZEN Blue (Zeiss). CZI files were read into HALO v2.3 (Indica Labs) for analysis. *GIPR* and *GLP1R* positive cells were detected using the HALO ISH v2.2 analysis module with a cell classifier trained to detect cells with classical neuronal morphology. Cells detected as positive for *GIPR* or *GLP1R* were checked by eye.

QUANTIFICATION AND STATISTICAL ANALYSIS

Data Analysis

Data are presented as mean and SD. Statistical analysis was performed using Microsoft Excel and GraphPad Prism 7.0. For all statistical tests, an α risk of 5% was used. Multiple comparisons were made using a 2-way ANOVA or a repeated measures 2-way ANOVA with a post-hoc Tukey or Sidak test, as indicated in the figure legends. Single comparisons were made using either a paired or unpaired Student's t tests where appropriate as indicated in the figure legends. N numbers represent the number of mice used in each study as indicated in the figure legends, with the exception of calcium imaging experiments, where n represents the number of cells imaged. For calcium imaging analysis, 78 *Gipr*-expressing cells from 16 separate preparations (representing cells isolated from 32 mice in total) were recorded.

DATA AND CODE AVAILABILITY

All raw scRNAseq data generated from *Gipr*-positive hypothalamic cells have been deposited into the NCBI GEO: database. The accession number for these data is NCBI GEO: GSE134726.

Cell Metabolism, Volume *30*

Supplemental Information

Glucose-Dependent Insulinotropic Polypeptide

Receptor-Expressing Cells

in the Hypothalamus Regulate Food Intake

Alice E. Adriaenssens, Emma K. Biggs, Tamana Darwish, John Tadross, Tanmay Sukthankar, Milind Girish, Joseph Polex-Wolf, Brain Y. Lam, Ilona Zvetkova, Warren Pan, Davide Chiarugi, Giles S.H. Yeo, Clemence Blouet, Fiona M. Gribble, and Frank Reimann

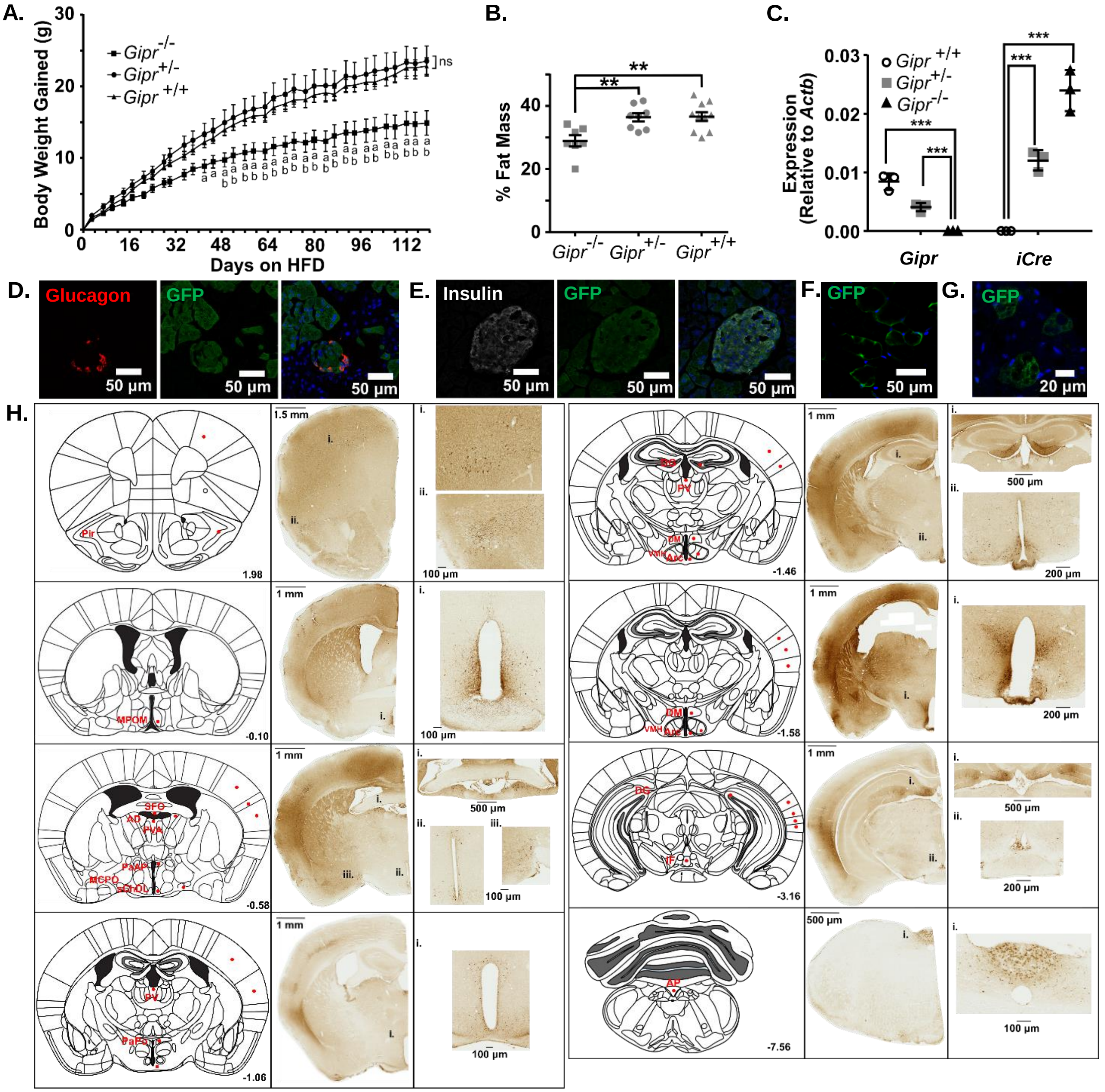

Figure S1: *Gipr*-Cre mice allow for the manipulation and identification of *Gipr*-expressing cells *in vivo* (Related to Figure 1)**:**

A. *Gipr*-Cre mice heterozygous for Cre at the *Gipr* locus were crossed producing offspring null, heterozygous, or homozygous for *Gipr*. Body weight was measured in response to 17 weeks on high fat diet. Significance in weight gain was tested via 2-way ANOVA with a Tukey's post hoc test; 'a' indicates $P < 0.05$ for *Gipr* $^{-/-}$ vs *Gipr* $^{+/-}$, 'b' indicates $P < 0.05$ *Gipr* $^{-/-}$ vs *Gipr* $^{+/+}$; n = 7 (*Gipr* $^{-/-}$), 8 (*Gipr* $^{+/-}$), 10 (*Gipr* $^{+/+}$). B. After 17 weeks on HFD, body composition was measured via NMR. Significance was determined using one-way ANOVA with a Tukey's post hoc test; n = 7 (*Gipr* $^{-/-}$), 8 (*Gipr* $^{+/-}$), 10 (*Gipr* $^{+/+}$). C. Relative expression of *iCre* and *Gipr* in whole islets isolated from *Gipr* $^{+/+}$, *Gipr* $^{+/-}$, *Gipr* $^{-/-}$ mice (n=3 for each genotype). Data are plotted as $2^{\Delta Ct}$ compared to *Actb* with bars representing mean +/- SD. Statistical significance was assessed through one-way ANOVA. Pancreas tissue from *Gipr*^{EYFP} mice was stained for GFP (green) and glucagon (red, D), or insulin (white, E). Inguinal white (F) and interscapular brown (G) adipose tissue from *Gipr*^{EYFP} mice was stained for GFP (green). H. Coronal sections from heterozygous *Gipr*^{EYFP} and heterozygous *Gipr*^{GCaMP3} mice were stained for GFP. Red circles represent the presence of GFP-immunoreactive somata. Drawings are based on the Paxinos Mouse Brain Atlas with the numerical values in the bottom right corner indicating the A/P location relative to Bregma. Pir, piriform cortex; MPOM, medial preoptic nucleus; SFO, subfornical organ; AD, anterodorsal thalamic nucleus; PVA/PV, paraventricular thalamic nucleus; PaAP/PaPo, paraventricular hypothalamic nucleus; MCPO, magnocellular preoptic nucleus; sChDL, suprachiasmatic nucleus; DG, dentate gyrus; ARC, arcuate nucleus; DM, dorsomedial hypothalamus; IF, interfasicular nucleus; AP, area postrema.

A.

	Snap25	*Sst*	*Avp*	*Pthlh*	*Slc32a1*	*Cartpt*	*Cnr1*	*Tac1*	*Slc17a6*	*Nts*	*Pomc*	*Htr2c*	*Unc13c*	*Cckbr*	*Ghsr*	*Th*	*Lepr*	*Npy*	*Mc4r*	*Agrp*
Snap25	229	189	133	114	100	97	89	75	63	20	19	9	8	8	3	3	3	2	2	0
Sst	189	197	123	105	94	97	83	62	48	17	18	5	9	8	3	3	3	2	1	0
Avp	133	123	141	68	70	63	62	49	40	13	14	5	9	7	3	2	3	2	1	0
Pthlh	114	105	68	117	77	69	56	40	19	11	10	1	3	2	0	3	1	0	0	0
Slc32a1	100	94	70	77	101	69	43	27	7	14	10	0	3	1	2	3	1	0	0	0
Cartpt	97	97	63	69	69	100	41	34	18	12	11	2	7	3	0	3	1	2	0	0
Cnr1	89	83	62	56	43	41	95	51	42	9	7	4	1	4	1	3	2	0	0	0
Tac1	75	62	49	40	27	34	51	80	45	6	7	8	4	4	0	1	1	1	1	0
Slc17a6	63	48	40	19	7	18	42	45	68	7	7	8	5	6	1	0	1	2	1	0
Nts	20	17	13	11	14	12	9	6	7	20	3	1	1	1	0	1	0	1	0	0
Pomc	19	18	14	10	10	11	7	7	7	3	19	0	3	2	1	1	0	1	0	0
Htr2c	9	5	5	1	0	2	4	8	8	1	0	9	1	0	0	0	1	0	1	0
Unc13c	8	9	9	3	3	7	1	4	5	1	3	1	9	2	0	0	0	2	0	0
Cckbr	8	8	7	2	1	3	4	4	6	1	2	0	2	8	0	0	0	2	0	0
Ghsr	3	3	3	0	2	0	1	0	1	0	1	0	0	0	3	0	0	0	0	0
Th	3	3	2	3	3	3	3	1	0	1	1	0	0	0	0	3	0	0	0	0
Lepr	3	3	3	1	1	1	2	1	1	0	0	1	0	0	0	0	3	0	1	0
Npy	2	2	2	0	0	2	0	1	2	1	1	0	2	2	0	0	0	2	0	0
Mc4r	2	1	1	0	0	0	0	1	1	0	0	1	0	0	0	0	1	0	2	0
Agrp	0	0	0	0	0	0	0	0	0	0	0	0	0	0	0	0	0	0	0	0

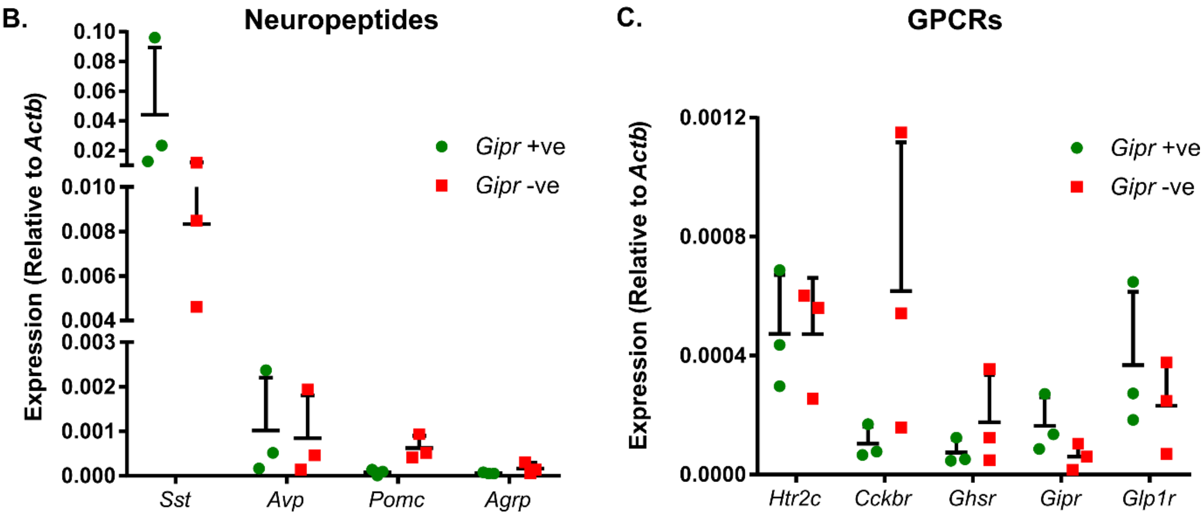

Figure S2. Expression of selected neuroendocrine targets in *Gipr* cells (Related to Figure 1)**:**
A. Expression matrix showing the number of cells from the neuronal cluster expressing a selection of neuroendocrine genes. RNA was extracted from FACS purified hypothalamic *Gipr*-expressing cells from heterozygous *Gipr*^{EYFP} or *Gipr*^{GCaMP3} mice and converted to cDNA. Gene expression of selected neuropeptides (B) and cell surface receptors (C) in *Gipr*-positive and *Gipr*-negative cells was measured by qPCR. Data are plotted as $2^{\Delta Ct}$ compared to *Actb* with bar representing mean + SD. n = 3 sorts, equivalent to 8 mice.

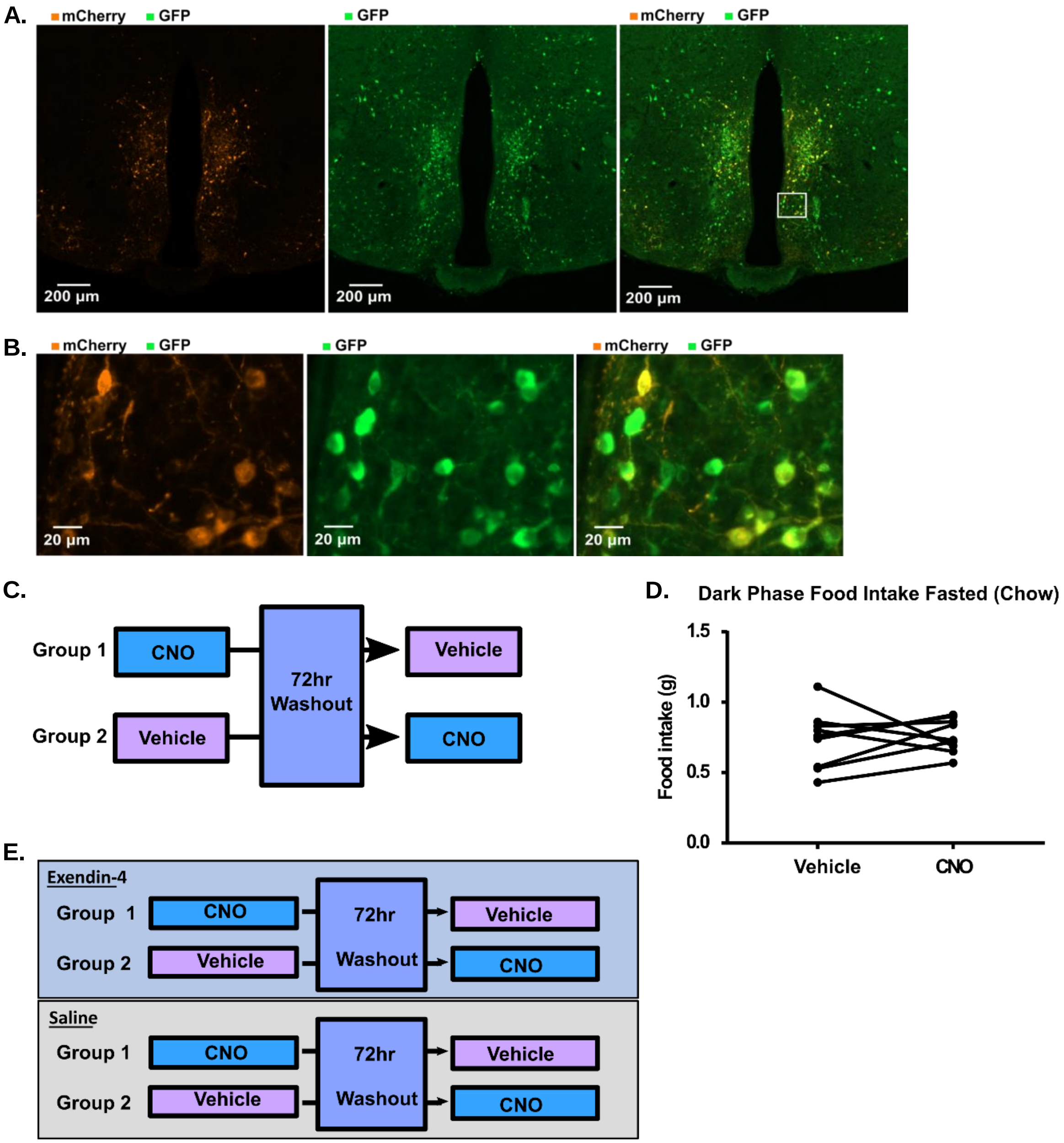

Figure S3: Targeting of AAV-DIO-hM3D-mCherry to *Gipr* cells; Food intake study design (Related to Figure 3,4)**:**
Brains were harvested from *Gipr*^{hypDq} mice. Coronal sections were stained for GFP and mCherry to assess the targeting of hM3D-mCherry into *Gipr*-expressing cells. A. Representative slice showing GFP (green) and mCherry (red) staining. B. Enlarged image of cells demarcated by white box in panel A. C. Illustration of crossover design used for experiments represented in Figure 3 and 4F. Each mouse served as its own control. D. Heterozygous *Gipr*-Cre mice that had not received injections of AAV-DIO-hM3D-mCherry were administered either CNO (1 mg/kg) or vehicle i.p following a 10hr daytime fast in a crossover design study. The amount of food consumed during the beginning of the dark phase was measured 2hr post-injection and compared using a paired t-test. n= 9. E. Illustration of crossover study design used for experiments represented in Figure 4G. Each mouse served as its own control.

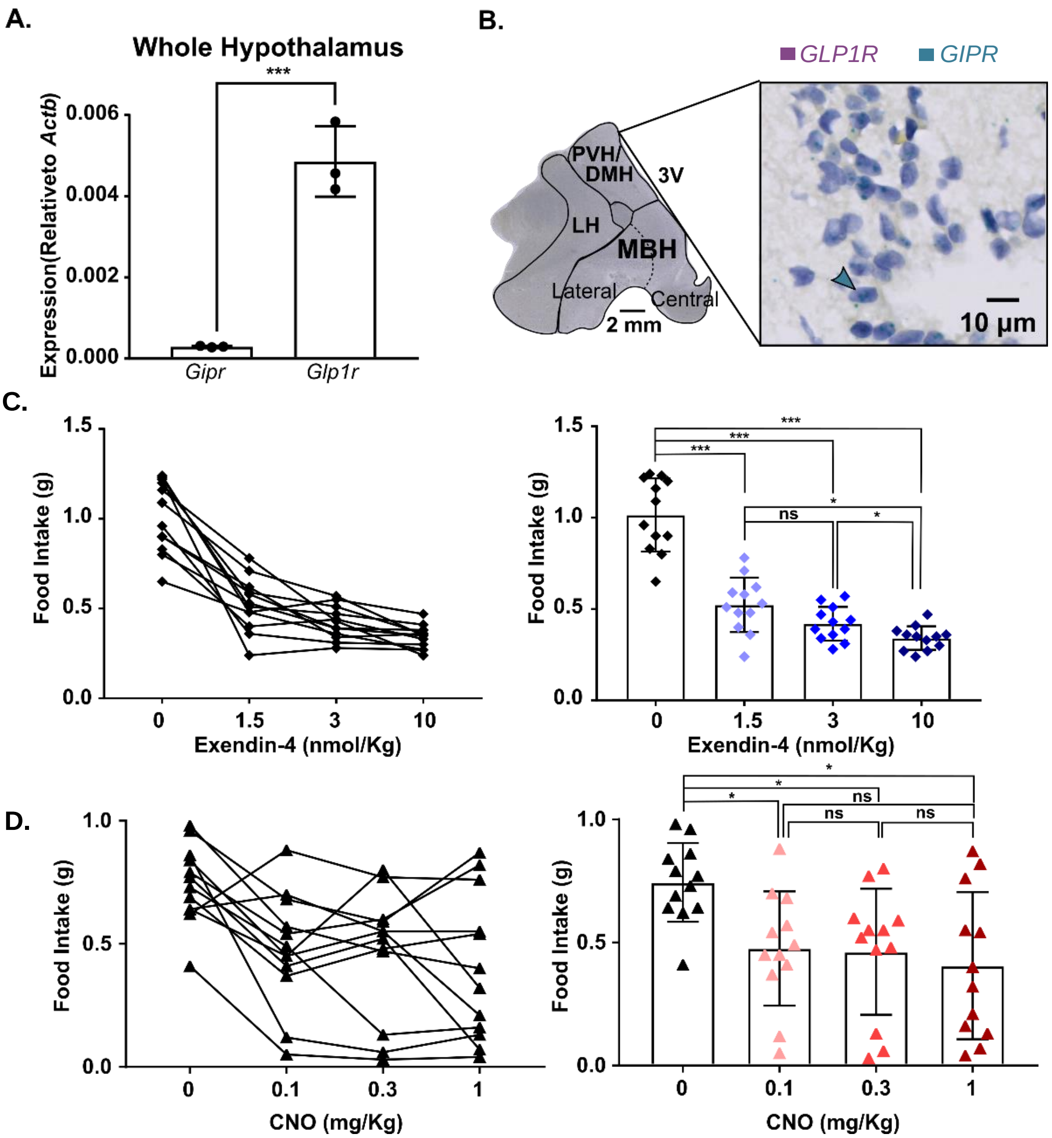

Figure S4: Hypothalamic *Gipr* localisation; CNO/Ex-4 dose response (Related to Figure 4)**:**
A. Relative expression of *Gipr* and *Glp1r* in whole hypothalamic homogenates in WT mice (n=3). Data are plotted as $2^{\Delta Ct}$ compared to *Actb* with bars representing mean +/- SD. Expression levels of *Gipr* and *Glp1r* were compared using an unpaired student's t test. ***P<0.001; n =3. B. Human hypothalamus tissue samples were labelled for *GIPR* and *GLP1R* mRNA using RNAscope. Cells lining the third ventricle (3V), likely to be ependymal cells expressing *GIPR*. C. Following a 10 h daytime fast heterozygous *Gipr*-Cre and *Gipr* WT mice were injected with Ex-4 (1.5, 3, or 10 nmol/kg) or saline s.c. 1hr prior to the onset of the dark phase. At the onset of the dark phase food was presented, and food intake measurements were taken 2hr later. This was a crossover design study where each mouse served as its own control. Food intake was compared using a repeated measures 2-way ANOVA with a Sidak's post hoc test. *P<0.05, ***P<0.001; n = 12. D. Following a 10hr daytime fast *Gipr*^{hypDq} mice were injected with saline s.c. 1hr prior to the onset of the dark phase. CNO (0.1, 0.3, or 1 mg/kg) or vehicle was injected i.p. at the onset of the dark phase, food was presented, and food intake measurements were taken 2hr post-activation. This was a crossover design study where each mouse served as its own control. Food intake was compared using a repeated measures 2-way ANOVA with a Sidak's post hoc test. *P<0.05; n = 12.
